# Algorithmic reconstruction of trophic networks from open-access species lists reveals key organisms in real ecosystems

**DOI:** 10.1371/journal.pcbi.1014061

**Published:** 2026-03-12

**Authors:** Miguel Brun-Usan, Roberto Latorre, Ángela D. Buscalioni, Paloma Alcorlo, Jesús Marugán-Lobón

**Affiliations:** 1 Departamento Paleobiología/ CIPb (Centre for the integration of Paleobiology). Universidad Autónoma de Madrid (UAM), Madrid, Spain; 2 Departamento Ingeniería Informática. Escuela Politécnica Superior. Universidad Autónoma de Madrid (UAM), Madrid, Spain; 3 Departamento Ecología. Facultad de Ciencias. Universidad Autónoma de Madrid (UAM), Madrid, Spain; 4 Centro de Investigación en Biodiversidad y Cambio Global (CIBC). Universidad Autónoma de Madrid (UAM), Madrid, Spain; University Hospital Schleswig-Holstein - Campus Kiel: Universitatsklinikum Schleswig-Holstein, GERMANY

## Abstract

Biotic interactions, crucial for understanding the ecology and evolution of species, are often conceptualized as ecological networks. However, the complexity of real ecosystems poses challenges for empirical inference, and theoretical interaction models, while informative, frequently fail to undergo empirical validation. This dual limitation creates a gap between theoretical and empirical approaches in portraying ecosystem dynamics and identifying (and protecting) key species, which are critical for conservation efforts and ecosystem management. In order to bridge this operational gap, we present a novel automated protocol capable of generating realistic trophic networks, including multilayer ones, using non-curated, freely-available species lists from real ecosystems as input data. As a proof-of-concept, we applied this method to the species lists contained in the RAMSAR database of wetland ecosystems. Our data mining algorithm enriches these species lists with functional traits, such as body size, habitat, and diet, by integrating information directly sourced from online biodiversity databases. Subsequently, a modified version of the Allometric Niche Model is used to sort species within the trophic network according to their functional traits and ecological roles. After demonstrating the algorithmic robustness of our method and the biological plausibility of the resulting ecological networks, we illustrate its potential to characterize, in a cost-efficient manner, the structure of real-world ecosystems and to identify the organisms that are crucial for maintaining that structure. In this case study, our findings indicate that the robustness of wetland ecosystems often depends on medium-sized, highly mobile organisms occupying intermediate trophic levels.

## Author summary

Understanding who eats whom in nature is key to conserving ecosystems, but inferring these complex food webs (trophic networks) in real environments is resource-intensive and sometimes unfeasible. Although theoretical models can generate plausible networks, they often lack direct links to real-world data. To address this, we developed a fully automated protocol that uses openly available species lists to reconstruct realistic ecological networks. Our method enriches these raw species lists with traits like body size, diet, and habitat, mined from public databases. Then, it applies a well-tested theoretical model to generate trophic networks where links represent biologically plausible interactions. We validate the networks against known ecological patterns and show that they capture the essential structural features of ecosystems. This approach offers a scalable, cost-efficient way to estimate interaction networks, identify keystone species, and assess ecosystem robustness, potentially informing biodiversity conservation and the management of vulnerable habitats worldwide.

## Introduction

The study of species interactions constitutes an active research field [1–3], and network-based representations are a powerful tool to this end [4]. However, species interactions are very complex, diverse and numerous (scaling to the square of the number of species), and characterizing ecosystems demands tremendous sampling efforts [4,5]. This posits a significant challenge for deducing the structure of real-world ecological networks and explains why publicly available databases (e.g., *Web-of-life*) often provide oversimplified representations of the real, functional ecosystems they aim to describe [6,7]. Despite such limitations, analyses of ecological networks have consistently revealed a highly structured architecture similar to that found in genetic, social or linguistic networks [8–12]. Such commonalities have paved the way to theoretical investigations where, by applying different algorithmic rules, *in silico* trophic networks with realistic topologies can be derived, enabling the simulation of eco-evolutionary dynamics [1,13–20].

However, the mismatch between algorithmically derived nodes in abstract networks and species in real-world ecosystems hinders the operational reconciliation between theoretical and empirical approaches [20,21]. A wide range of methods has been developed to estimate ecological networks, including trait- and allometry-based approaches [22], proxy-based inference of biotic interactions [5], and niche- or size-structured food-web models linking foraging behavior to network structure [23]. Despite these advances, many existing models rely on abstract functional groupings rather than explicitly resolved species, which can limit their applicability to empirical systems [24–25].

A potential strategy to solve this situation relies on the development of automated methods capable of estimating networks of species interactions in a realistic, efficient and standardized manner, based on accessible data such as online species lists [5,22]. Such procedures would be well-suited for bridging the application of theoretical ecology and network models to real-world case-studies, including the conservation of endangered habitats or the reconstruction of extinct ecosystems. Approaches following this logic have been proposed for restricted scenarios (e.g., fisheries, [22,26]), yet a general procedure to automatically resolve large-scale ecological networks is still lacking [27].

**Fig 1:**
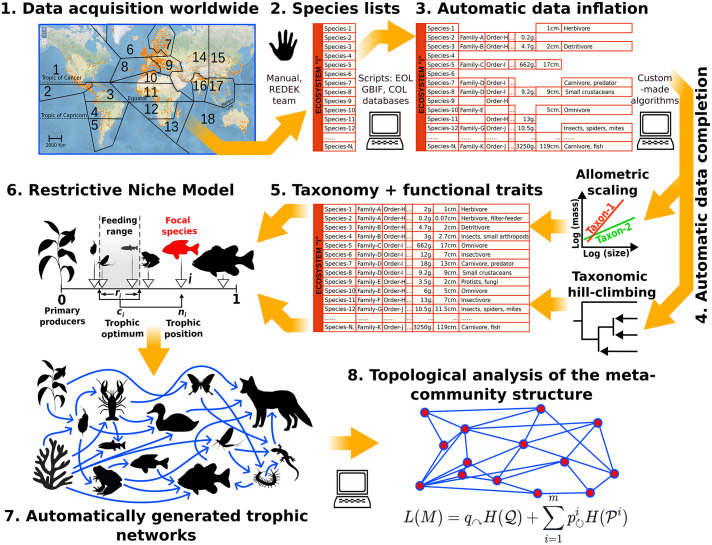
Conceptual depiction of the proposed automated protocol. 1-2. Inputs consist of non-curated species lists. In our case study, we compiled lists from ≈160 RAMSAR wetland communities worldwide. 3. These raw species lists were enriched with functional traits (e.g., size, diet) automatically mined from public databases. 4-5. Missing data were estimated using two complementary strategies: (i) previously known allometric body mass/size relationships, and (ii) taxonomic inference, where missing values were inferred from the closest phylogenetic relatives. 6. The resulting enriched lists containing trait values were introduced as input parameters in the allometric niche model, a theoretical method capable of generating realistic food webs. 7. In the resulting (automatically constructed) networks, nodes correspond to the species found in the original ecosystem, and links represent highly plausible trophic interactions. 8. This procedure can create large ensemble of homogeneous and realistic trophic networks amenable for quantitative and comparative analysis. Credits: Panel 1 map: Panel 1 world map showing Ramsar sites sourced from the Ramsar Sites Information Service (RSIS, rsis.ramsar.org), licensed under Creative Commons Attribution 4.0 International (CC BY 4.0). Animal silhouettes in other panels were obtained from OpenClipart (openclipart.org), which releases all content under CC0 (public domain).

Here, we present a general automated protocol for generating realistic trophic networks, including multilayer ones, based on raw, open access species lists. Our method utilizes automatically recorded functional attributes of species as a bridge between theoretical models and real-world ecosystems, allowing for a massive generation of ecological networks and serial data analysis (see Fig 1 for a schematic conceptualization). To illustrate the ability of the method to address features of real-world ecosystems, such as spatial compartmentalization, temporal dynamics and high biodiversity indexes [28], we apply it to the RAMSAR database, an online resource of species composition of wetlands for their conservation and sustainable management. We describe first the algorithm and statistically demonstrate its robustness and biological plausibility. Later, demonstrate its ability to identify functional modules and key species, contributing to the development of practical solutions and guide best practices in applied ecology.

## Results

The Results are structured into two blocks. The first aims to validate the protocol by evaluating its performance in capturing features of real-world ecosystems. The second analyzes its capacity to recover key ecological features under different scenarios, some of which are highly relevant to conservation ecology. The quantitative descriptors used for this purpose, including their acronyms and brief definitions, are summarized in Box 1, which serves as a quick reference for conceptual and computational details.

### Validating the model I: Automatically constructed networks are structurally realistic

**Fig 2:**
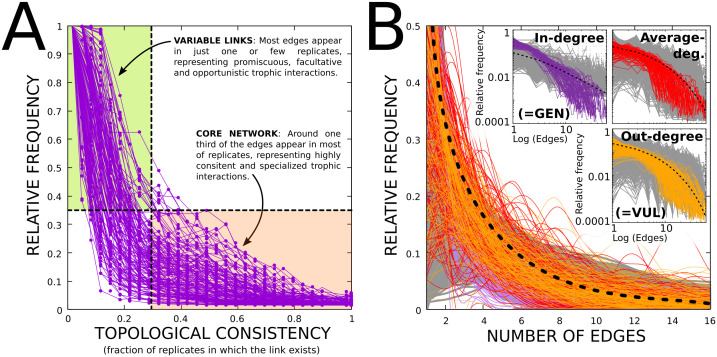
(A). Topological consistency (TC, X-axis) denotes the fraction of replicates of a given ecosystem in which a specific trophic interaction exists. A large TC indicates highly stable interactions. The Y-axis represents the relative frequency of links (within a given replicate) that exhibit certain TC. As the Figure shows, although most links vary across replicates (facultative interactions), a core, stable sub-network of highly consistent links (specialized necessary interactions) remains stable. Each line represents one replicate of a given ecosystem. **(B)**. The automatically constructed trophic networks show a scale-free degree distribution (DEG). X-axis: number of trophic interactions per node. Y-axis: relative frequency of species having that degree (within a replicate). Line colors as in the (Log scale) insets. Red: all interactions. Purple: only-input interactions (GENnerality). Orange: only-output interactions (VULnerability). Grey and dashed lines: individual (1000) replicates and average distributions under the pure ANM. Insets use logarithmic axes (log-scaled, not log-transformed data) to highlight the scale-free-like structure of the distribution.

To test whether our algorithm (see Materials and Methods) can generate biologically plausible trophic networks from unstructured species lists, we first examined whether the topology of the simulated networks reproduced the characteristic scale-free architecture observed in empirical food webs. This architecture can be detected by analysing the distribution of node degrees, that is, the probability that a node has *k* trophic interactions (DEG; see Box 1). In real ecosystems, only a small number of species participate in many trophic interactions (corresponding to highly connected nodes in the network representation), whereas the majority of species engage in relatively few interactions [15–17]. This structural heterogeneity gives rise to a heavy-tailed *degree distribution* DEG, which is commonly well described by a power-law or, in some cases, a negative exponential distribution [10,12]. As shown in Fig 2B, the degree distribution of our simulated networks closely follows an approximate power-law of the form DEG(*k*) ∝ *k*^−γ^, indicating a scale-free architecture. This pattern holds not only for the overall network but also when links are analysed according to their direction; that is, considering only the outgoing links from each species to its consumers (consumer vulnerability, VUL) and the incoming links to each species from its resources (resource generality, GEN) [15,29,30], as shown in the insets of Fig 2B.

**Fig 3:**
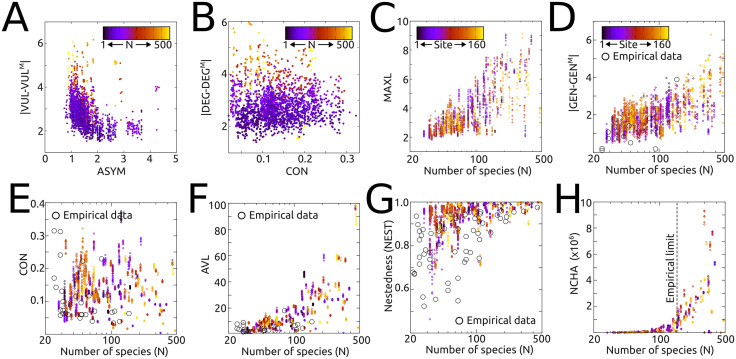
Representative two-dimensional projections of the multivariate network space defined by the quantitative descriptors summarized in Box 1. Each point corresponds to one automatically generated network (one ecosystem × one replicate). When available, data (represented as empty black circles) corresponding to empirically measured properties of real food webs (mainly from refs. [29,32]) are also shown for comparison. **(A)** In-degree/out-degree asymmetry vs. absolute deviation in vulnerability relative to the pure allometric niche model (denoted by the M superscript). **(B)** Network connectivity vs. absolute deviation in total degree relative to the pure niche model. **(C)** Species richness vs. maximum number of trophic interactions involving a single species. **(D)** Species richness vs. absolute deviation in generality relative to the pure niche model. **(E)** Species richness vs. network connectivity. **(F)** Species richness vs. average number of trophic links per species. **(G)** Species richness vs. network nestedness. **(H)** Species richness vs. number of distinct trophic chains in the network. In panels A and B, colors encode species richness (N) for the corresponding network. In panels C-H, colors are used to identify the ecosystem of origin (from 1 to 160) to visually group replicates belonging to the same site.

After establishing that the generated networks reproduce the scale-free organization characteristic of empirical food webs, we next assessed their structural realism using a multivariate morphospace analysis [10,12], which allows multiple quantitative topological network descriptors to be evaluated simultaneously. Following an unbiased approach, we based the analysis on the full set of 30 quantitative network descriptors listed in Box 1, thereby defining a 30-dimensional abstract network space populated by about 3000 data points (ecosystems × replicates). As shown by representative two-dimensional projections of this space (Fig 3A–3H), the distribution of networks in the morphospace is highly non-random and anisotropic: most networks cluster within a relatively small hypervolume, while large regions of the theoretically possible space remain unoccupied. This restricted occupation indicates that our algorithm generates a limited subset of possible network topologies, a property commonly associated with functional networks [10,12,27,31].

When empirical data were available, the morphospace analysis also allowed direct comparison between the properties of our networks and those measured in real food webs (mainly taken from references [21,29] and [32]). In these cases, the values of the corresponding network descriptors generally fell within the empirically observed ranges (see empty, black circles in Fig 3D-3G). Importantly, following previous studies [29,30], we compared our network attributes with those obtained under the “pure” allometric niche model (ANM), that is, in the absence of additional functional constraints. These deviations (often termed model error, ME) were used, as in the aforementioned works, both as quantitative descriptors *per se* and as a reference baseline. These ME-based comparisons show, first, that the resulting MEs are slightly larger but of comparable magnitude to those reported in previous studies comparing empirically derived food webs with the ANM. Importantly, the relevance of these deviations (also visible in Fig 2B) is limited, as the goal of our approach is not to maximize agreement with the pure ANM but rather to reproduce the structural properties of real ecological networks, an aspect that is explicitly evaluated in the following sections. Second, our comparisons reveal that incorporating functional constraints in the establishment of trophic interactions not only reduces interaction density but also systematically modifies several key structural properties of the resulting networks relative to the ANM baseline, indicating that, although our approach is strongly grounded in the ANM, it is not algorithmically reducible to it.

Finally, it is important to note that, because of sampling limitations, topological features of empirically derived networks (including those used here), have largely been restricted to relatively small networks, rarely exceeding species richness values of *N* ≲ 100 [21,29,32]. As illustrated in Fig 3, our approach enables the exploration of network attributes in much larger ecosystems, providing robust estimates of their expected topological properties. In this context, our analysis confirms that previously reported trends and patterns (such as the non-linear increase in average trophic chain length and in the number of trophic chains with species richness [33,34]) also hold across this extended range of network sizes, which has so far remained empirically inaccessible (Fig 3H).

### Validating the model II: Simulated networks contain realistic predator-prey interactions

**Fig 4:**
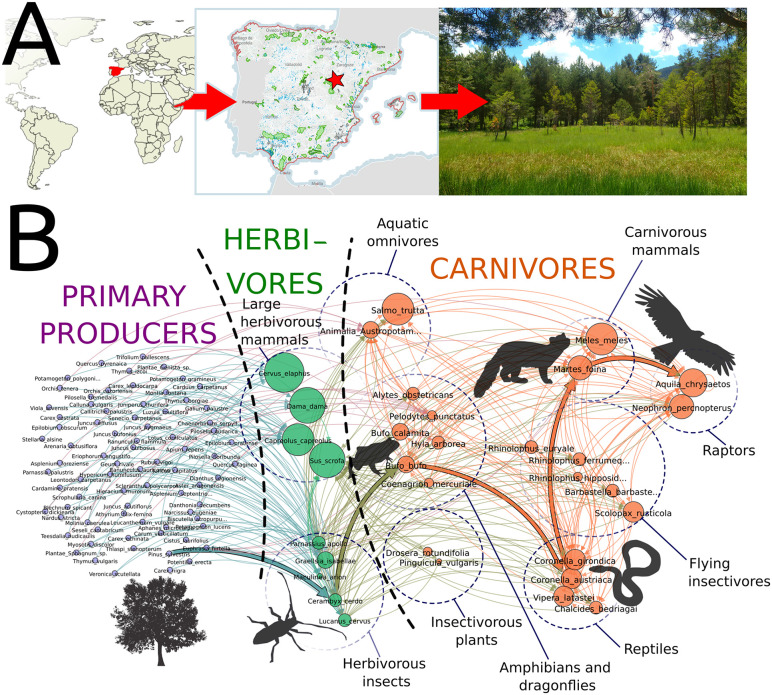
Example of a biologically plausible trophic network automatically generated by our algorithm. **(A)**. Case example: the RAMSAR wetland Orihuela del Tremedal (Spain) whose original species list (N = 110) included un-resolved names. **(B)**. An example (1^st^ replicate) network reveals that simulated trophic interactions are generally consistent with the known lifestyles of the species present in the ecosystem. For illustrative purposes, the silhouettes of representative species of the main trophic levels have been juxtaposed to the network. Node size reflects relative body mass; while colors indicate functional guilds (blue = plants, green = herbivores, orange = carnivores). Notice that some highly specific relationships such as insect-eater plants (circle in the central, lower part of the Figure), are adequately captured by the automated algorithm. This is achieved by introducing a few algorithmic rules devoted to handle these highly specific interactions (see Materials and Methods and SI). Credits: Panel A (left to right): world map from Natural Earth (naturalearthdata.com, public domain); map of Spain from the Instituto Geográfico Nacional (IGN), licensed under Creative Commons Attribution 4.0 International (CC BY 4.0); original photograph taken by M. Brun-Usan. Panel B: animal silhouettes obtained from OpenClipart (openclipart.org) and released under the CC0 (public domain) dedication.

Beyond apparent or structural similarities with empirica food webs, we assessed whether the specific trophic links generated by our algorithm actually correspond to empirically recorded interactions. As an initial qualitative validation, we manually inspected a sub-set of the > 3000 generated networks (replicates 1 and 2 of all sites) to determine whether the position of their nodes was consistent with the lifestyle of the represented species. We found that the algorithm consistently sorts the species according to the trophic position that they occupy in nature, reflecting what could be recorded under direct observation (see Fig 4 for a representative example). Interestingly, due to algorithmic stochasticity, and that the *c*_*i*_ and *r*_*i*_ parameters of the allometric niche model are randomly drawn from uniform (U) and β distributions (see Methods), each different run for the same RAMSAR wetland produced distinct networks. A closer analysis shows that indeed only a “core subnetwork” (≈30%) of interactions remains relatively constant across replicates, while >50% of the edges vary (Fig 2A). A sensible interpretation of this result is that our algorithm does not produce a network in which each species is fully connected to everything it can potentially eat, as classical food web representations do. Instead, each replicate shows a “snapshot” of what species might be *potentially* feeding on at a given time, analogous to a field sample (see *Discussion* for detailed treatment of *potentiality*). In other words, the networks resulting from our approach do not represent the cumulative record of all possible interactions for each species, nor do they restrict species to a single interaction per replicate; rather, each replicate draws a subset of interactions that a species (or an individual) could experience within a discrete timeframe, sampled from the species’ full ecological potential. This effectively mirrors the balance between deterministic and stochastic processes observed in the biological realm. On one hand, the stable core reflects highly deterministic, obligate interactions driven by trophic specialization and other functional constraints. Within our framework, such a stable core of interactions emerges from the feeding preferences recorded for specialist taxa, which impose a highly stringent filter on potential links. As a consequence, even across replicates with varying parameterizations of the allometric niche model, only a limited and consistent set of interactions is realized. In contrast, the variable fraction of links captures stochastic influences such as opportunistic feeding, transient prey availability, seasonality, or simple chance, which can lead species to feed on different prey in different spatiotemporal contexts. Importantly, this result is not only conceptually relevant, but also allows for more detailed identification of key species and functional groups within ecosystems, with implications for conservation biology (see below and Discussion).

**Fig 5:**
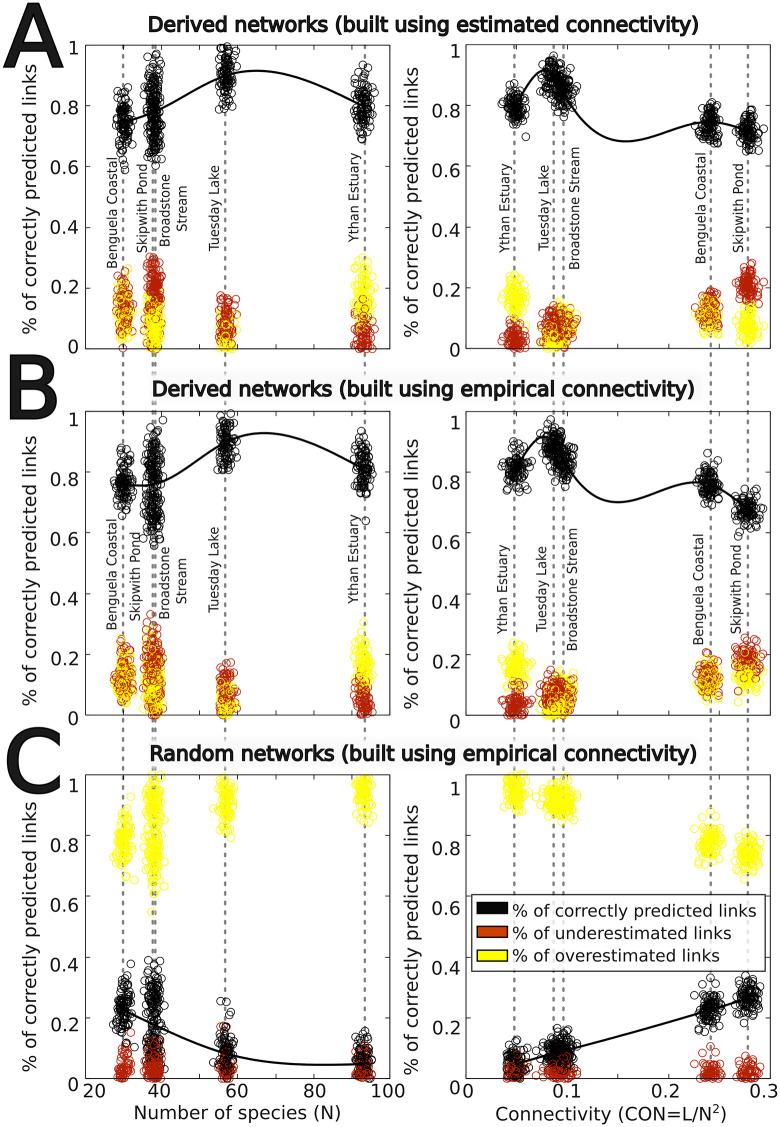
Quantitative comparison between empirically measured trophic interactions and those predicted by the automated protocol. The Figure compares, on a link-by-link basis, 100 automatically generated network replicates with the corresponding empirical food webs reported for five wetland-like ecosystems with varying species richness and connectivity [6]: Benguela Coastal Ecosystem (N = 29), Ythan Estuary (N = 92), Tuesday Lake (N = 56), Skipwith Pond (N = 37), and Broadstone Stream (N = 37). Underestimated links (red) correspond to interactions present in the empirical network but missing in the reconstructed networks, whereas overestimated links (yellow) are interactions present in the reconstructed networks but absent from the empirical data. **(A)** Comparison using a default estimated average connectivity of ≈0.12. **(B)** Comparison using the empirically measured connectivity for each ecosystem. **(C)** Comparison with equally connected random networks, in which each possible link is realized with a probability equal to the empirical connectivity.

To conduct a more rigorous, quantitative test, we next used the basic setting of our protocol to generate 100 virtual replicates of five well-resolved and widely referenced empirical networks found in the literature [6], and checked, one by one, if the predicted interactions were present in the reference (empirical) networks. An interaction (or its absence) was considered correctly predicted only when both the interacting species and the direction of the interaction matched the empirical record. As shown in Fig 5A, our protocol typically predicts over 80% of the empirically recorded interactions, and that performance is relatively insensitive to the species richness or overall network connectivity. A closer inspection of the interactions that are not correctly predicted by the model shows that they are not fully attributable to errors of the model, but to the assumption of shared habitat, namely, that any two organisms satisfying the allometric and trophic criteria can potentially interact. For example, if a species list includes both a marine dolphin (carnivore) and a rabbit, having an adequate allometric relationship, the basic algorithm may allow a potential interaction despite their spatial segregation. Fortunately, those exceptions to this rule can be easily handled using different buffering strategies (see SI and the section on Multilayer Networks below).

Finally, to assess whether the level of agreement could arise by chance, we compared our results with equally connected random networks. In this case, prediction accuracy dropped to approximately 20% (Fig 5C). This marked contrast between random and reconstructed networks demonstrates that the correspondence between predicted and empirical interactions reflects genuine biological signal rather than a statistical artifact.

### Identifying key organisms for maintaining ecosystem structure

**Fig 6:**
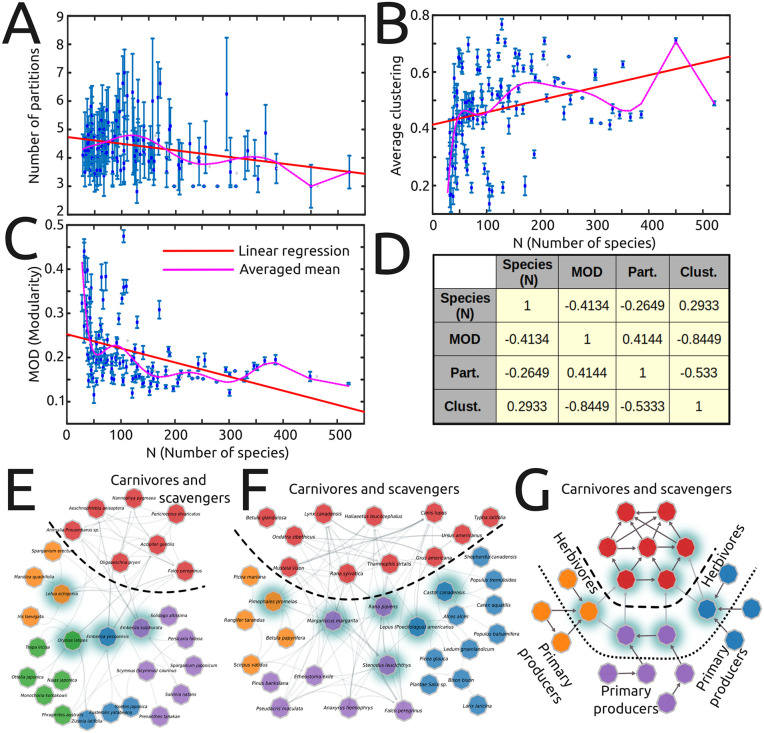
Modularity analysis. **(A)**. The number of semi-independent modules remains relatively low (around 4) and constant for most ecosystems. **(B)**. The average clustering (CLUST) increases in large ecosystems. **(C)**. The average modularity (MOD) decreases in large ecosystems. **(D)**. Some of the modularity-related measures are linearly correlated as indicated by the corresponding Pearson’s correlation coefficient. Anticorrelation between CLUST and MOD suggests that modules become less independent as ecosystems grow in size. **(E-F)**. Two case examples of modular structure in our dataset: **(E)**. Naikaikemi (Japan, N = 28), and **(F)**. Whooping Crane (USA, N = 36). Colors represent the optimal division maximizing modularity, dashed line separates carnivores-scavengers from the remaining organisms and dashed nodes represent “bridge” species between modules. **(G)**. An idealized network showing how the modules are, in general, organized across networks.

Modularity analysis (based on the Louvain method, see SI) reveals positive modularity in all simulated networks (0.1 < MOD < 0.5, Fig 6A), indicating the existence of functional subgroups (communities). These values are fully consistent with the few available estimates of modularity in empirical food webs (e.g., [35]), which typically report MOD values in the range of ≈0.2-0.4. Detailed inspection of these communities further reveals that the most densely (intra-connected) modules tend to be enriched with carnivores, while the remaining communities show a more hierarchical and sparser structure, composed by cohorts of functionally similar herbivores and the plants they preferably consume (Fig 6E-6G). This structure roughly corresponds to that typically found in actual ecosystems (Fig 6G), where herbivores engage in specific interactions due to plant defences (i.e., toxins) [24,36,37]. In this context, modularity provides a heuristic framework for identifying species that are critical for maintaining ecosystem connectivity and structure (in the sense that their extinction may elicit a fragmentation of the whole network, triggering extinction cascades and a loss of ecosystem functioning). According to network theory [10,15], those species are not necessarily those with the highest degrees, but rather those acting as functional links between modules. Our analysis reveals that those key species acting as “bridges” between modules tend to be medium-sized, high-mobility, generalist animals, such as frogs, rodents or crayfish that feed in one ecological compartment (e.g., water) but serve as prey in others (e.g., land) (Fig 6E-6G).

Additionally, the high modularity (MOD) values observed in smaller ecosystems (N < 150) and the negative correlation between MOD and the number of partitions PART further suggest that, while the number of modules remains relatively constant (around PART = 4) across ecosystems (Fig 6A and 6D, similar to empirical values reported in [35]), the modules become less independent as the number of species increases (Fig 6B-6C). This is also evidenced by a consistent increase in the average clustering CLUST, which indicates that inter-module connections become denser in larger networks (Fig 6D). This scaling implies that ecosystem connectivity increasingly relies on a growing set of bridge species, whose loss may disproportionately affect network integrity and functioning. In other words, larger ecosystems may require more key bridge species to maintain their structure and ecological functions, with clear implications for ecosystem management (see Discussion).

### Contrasting the ability of functionality-based and taxonomy-based sampling methods in capturing ecosystem structure

Since sampling resources in field surveys and monitoring programs are often limited, we asked if our method could help identify which ecosystem features should be measured to best capture its structure with minimal effort. To address this, we performed a set of rarefaction experiments that simulate two common sources of input uncertainty: loss of taxonomic resolution and incomplete sampling. In each case, we applied controlled perturbations to the input data and compared the resulting networks with the original (i.e., unperturbed and thus less noisy) ones.

**Fig 7:**
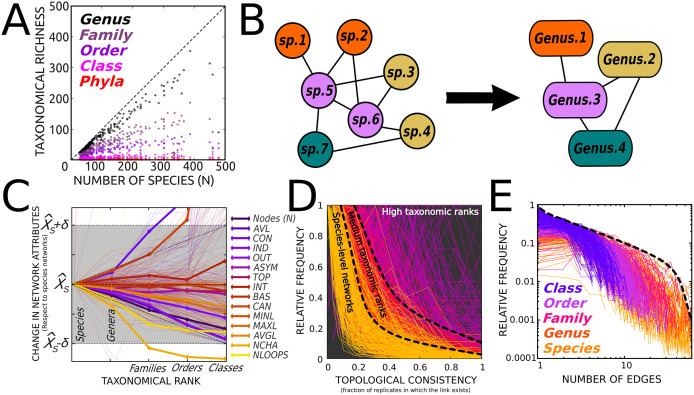
Robustness to loss of taxonomical resolution. **(A)**. Relationship between species richness and taxonomic richness across ecosystems, shown for multiple hierarchical levels (number of genera, families, orders, classes, and phyla). Each point corresponds to an ecosystem. This panel shows how the number of different genera in the ecosystems (each representing functionally similar organisms) escalates in proportion to the number of species N, which helps explain why our method is relatively insensitive to moderate taxonomic rarefactions (see next panels). **(B)** Taxonomic rarefactions were introduced by progressively collapsing species into higher taxonomic ranks (species → genus → family → order), producing genus-, family- and order-levels nodes. The panel illustrates how species belonging to the same genus (same color) in a schematic species-level network collapse into a single node in the corresponding genus-level network. For each collapsed node, body size was set to the mean of member species, and diet/habitat were aggregated as the union of their categories. After each taxonomic rarefaction, the trophic networks were recalculated, and their topological features compared with the species-level network. **(C)**. Most topological descriptors remain, at least up to the family rank, within certain confidence interval (defined as the mean ($\hat{X}$) ± the variance (σ) of that descriptor in the species-level network). **(D)**. Many of the links in the networks constructed using medium taxonomic ranks (orange) remain similar to the species-level networks (yellow lines and Fig 2A). **(E)**. Many above-species networks retain the scale-free architecture of the original, species-level networks (yellow lines and Fig 2B).

As a first step, we characterized how species richness relates to taxonomic richness across ecosystems in our dataset (Fig 7A). Species richness showed a regular and predictable scaling with the number of genera, families, orders, classes and phyla, reflecting the progressive aggregation of species into higher taxonomic ranks. Although taxonomic categories are not defined on functional grounds, this systematic relationship provides an empirical basis for assessing how a poor taxonomic resolution (that occur in many available species lists) may affect the quality and realism of the reconstructed trophic networks. To that end, new input lists were generated by grouping the species into progressively higher taxonomic ranks whose size and diet/habitat attributes were, respectively, the average and the aggregate of all different values in the one-rank-below list (Fig 7B). As shown in Fig 7C, most topological descriptors remained within acceptable bounds up to at least the family level, as quantified by the absolute standardized difference (|$\hat{X}$_S_ $\hat{X}$| / σ). Beyond global descriptors, link-level consistency was also high, with comparable occurrence-frequency distributions at the species level and in intermediate-rank networks (Fig 7D). Finally, the degree distribution preserved a heavy-tailed shape consistent with a scale-free architecture (Fig 7E).

**Fig 8:**
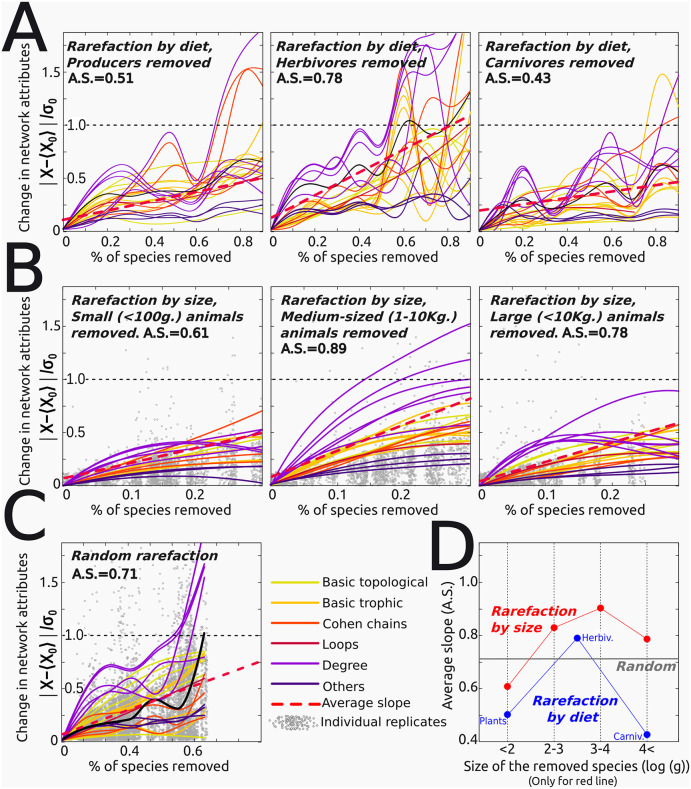
Robustness to functional rarefactions. Y-axes in panels A–C represent the absolute difference between each measured network attribute and the mean value of the corresponding attribute in the original (unperturbed) networks, expressed in units of the standard deviation of that attribute across replicates of the original networks. Accordingly, values close to zero indicate negligible differences, values around one indicate deviations comparable to the intrinsic variability of the original networks, and values greater than one indicate differences exceeding that variability. **(A)**. The ecosystems’ structure is more deeply affected when herbivores are removed, while removing plants or carnivores has a milder effect. However, the removal of any trophic group has catastrophic effects on the network structure if >N/2 of species are removed (see Figs 7, S2 and panel **(C)**). **(B)**. The ecosystems’ structure is deeply affected when medium-sized organisms are removed. As the four size intervals defined do not cover all (>12) orders of magnitude present in the whole dataset, the % of species removed (X-axis) never reaches the N/2 limit as in **A. (D)**. A summary of the general trends shown in previous panels taking the average slope (A.S.) as a proxy of network change. Most topological attributes vary more intensely, and above a random rarefaction **(C)**, when herbivores and/or medium-sized animals are removed. In **(B-C)**, grey points correspond to individual (perturbed) replicates. Red dashed lines: linear regressions of the represented data (with slope **A.**S.).

Together, these results justify, for instance, the use of genus-only lists as a reliable proxy in functional ecology studies. Furthermore, rarefaction experiments also show that datasets containing >50% of the original species sampled suffice to capture the structure of most real-world ecosystems, as the resulting networks are structurally similar to the fully informed network. However, substantial differences in the properties related to degree distributions (DEG, VUL, GEN; Box 1), advise caution (Fig 8C).

To analyse whether functional groups (defined based on functional traits) are more or less important than taxonomy in capturing the structure and identity of ecosystems, we performed a set of targeted perturbations where rarefaction was applied to functional subsets of species in the datasets [38]. We defined two different (discrete) partitions, one according to diet (Fig 8A), and another according to body mass (10^1^ to 10^5^g., Fig 8B). Then, 90% of the species were randomly removed in each functional group, and the structure of the resulting networks compared with that of the unperturbed network.

Results of these experiments show that removing plants causes a more unspecific and denser rearrangement of plant-herbivore interactions (as it happens in temperate regions during winter), while removing carnivores makes the remaining ones more generalist. In contrast, topological attributes vary significantly (i.e., above the baseline defined by random perturbations) when herbivores or medium-sized animals are removed, suggesting these organisms may play a key role in maintaining the ecosystems’ structure (Figs 8D, S2).

### Multilayer networks as potential tools for conservation prioritization

Because of spatial, physiological or phenological barriers, most organisms only interact with those that share the same ecological compartment [2,36,39]. Additionally, species often display different ecological roles across life stages or sexes and engage in various types of interactions beyond trophic ones. These complexities are hard to capture using “classic networks” [28], limiting their ability to identify the key biotic processes. A promising solution which is gaining momentum in recent years is to use a “multilayer” formalism where the basic trophic networks are unfolded in abstract spaces to account for different biological processes [40].

Here, we consider: 1) whether the construction of multilayer networks is also amenable to automatization, and 2) whether such multi-layer approach can be potentially useful at informing conservation strategies. As a proof of concept, we used a refined version of our protocol (see Methods) to automatically construct multilayer networks where one extra dimension corresponded to ontogeny and the other to habitat compartmentalization.

**Fig 9:**
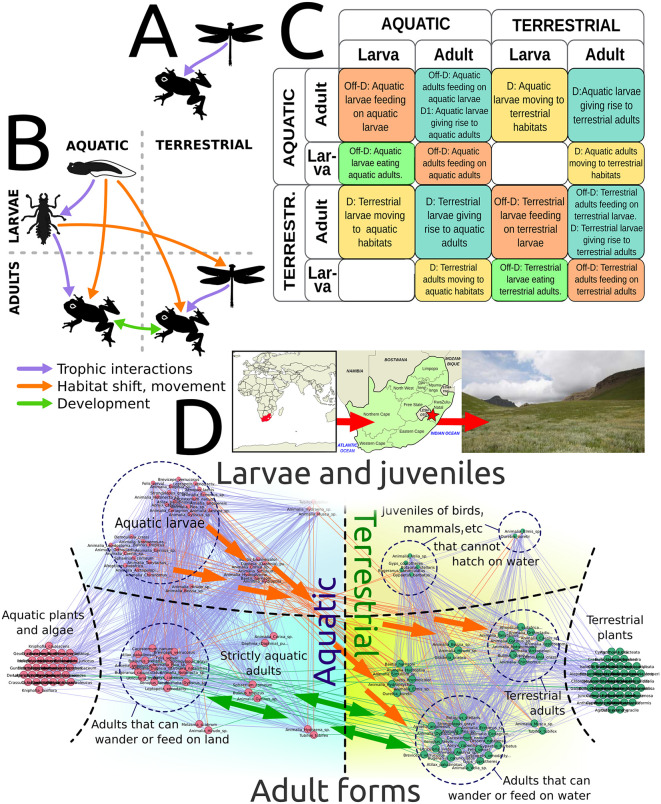
Multilayer networks. **(A)**. Classical networks cannot capture the complex interactions of amphibious/biphasic life cycles. **(B)**. A multilayer network with larval-adult and aquatic-terrestrial compartments allows representing, for instance, adult frogs feeding on adult dragonflies and dragonflies’ nymphs feeding on frogs’ tadpoles. **(C)**. Organisms movements across layers depend on key functional traits that can be mined from public databases. These traits can inform rules to automatically build multilayer networks via a matrix operator **(D)**. An automatically constructed multilayer network (Ntsikeni Reserve, South Africa). Most trophic edges (purple) occur within sub-habitat compartments, yet some adult organisms can move and feed across sub-habitats (green bi-directional arrows). Many ontogenic edges (orange) connect the aquatic larvae with terrestrial adults (e.g., dragonflies). Big arrows and dashed circles have been manually added to show the main functional groups and their connections. N = 170, half of the plants removed for the sake of clarity. Credits: Panels A-B: animal silhouettes obtained from OpenClipart (openclipart.org), released under the CC0 (public domain) dedication. Panel D (left to right): world map from Natural Earth (naturalearthdata.com, public domain); map of South Africa from Natural Earth (naturalearthdata.com, public domain); photograph from RAMSAR (rsis.ramsar.org), licensed under Creative Commons Attribution 4.0 International (CC BY 4.0).

The application of the protocol to a subsample of ecosystems resulted in a number of automatically-constructed multilayer networks where trophic interactions appeared as within-layer edges, while movement and development were represented by inter-layer edges. Crucially, the trophic and non-trophic links in those networks were well aligned with the known life histories of the organisms (Fig 9C). Although no ecosystem-level information was contained in the original species lists, well-documented ecological strategies naturally emerged as dense bundles of directional edges (e.g., *r*-strategist organisms developing in high-energy aquatic environments and giving rise to adults that can feed in water and land alike, [36], Fig 9D); suggesting that a few simple rules can effectively capture the deep, entangled structure of real ecosystems. Importantly, the multilayer approach uncovers significant biomass transfers not related to grazing or predation, which can be important for designing conservation strategies. For instance, it underscores the importance of some organisms such as amphibians, holometabolous insects, and vertebrates whose adults feed on both terrestrial and aquatic preys (Fig 9D) in connecting the different sub-habitats within spatially complex ecosystems like the studied wetlands.

Although the dense bundles of interactions revealed by our multilayer representation naturally suggest a modular organization of ecosystem processes, applying formal modularity analyses to these multilayer networks is not appropriate in our case. Current approaches to multilayer modularity remain methodologically limited and lack the level of consensus achieved by single-layer methods, relying, for instance, on random walks [41], spectral analyses [42], or higher-order harmonic motifs [43], each yielding disparate partitions that are difficult to reconcile and interpret biologically. Furthermore, identifying the species that connect ecological compartments (e.g., water–terrestrial or larval–adult layers) is sufficient for our purposes and already provides an intuitive indication of their ecological importance, which might be further supported in future work by considering taxon-specific biomass transfers across compartments.

## Discussion

We present a cost-effective and algorithmically robust method that efficiently transforms readily available, non-curated species lists from real-world, structurally complex ecosystems into highly realistic trophic (and multilayer) networks with up to 80% similarity to empirical ones. This approach not only significantly expands the pool of available networks for comparative studies but also enables the application of theoretical ecology and network theory to the actual species that constitute the targets of conservation policies. Indeed, our preliminary investigations around the method itself have yielded valuable insights of broad interest for ecologists and conservation biologists. First, rarefaction experiments indicated that an exhaustively taxonomic approach might not necessarily be the best strategy to characterize an ecosystem. This is because species within genera, and genera within families are functionally similar, while higher ranks may contain very different species only related by their internal anatomy and bodyplan (i.e., phylogeny) [44]. Indeed, many real-world databases pay relatively little attention to exact taxonomic resolution and often mix “true” species with higher-level categories, such as genera or families, or even with vernacular categories like “tadpole” or “earthworm.” Although this practice introduces mismatches between the number of taxa and the number of nodes (species richness, N) in the resulting networks (see Table C in S1 Text), our analysis shows that taxonomic resolution has little impact on the construction of trophic networks, making such approximations generally acceptable. However, what appears more essential is to diversify the sampling efforts across different functional groups, including representatives of the micro- and mesocosmic scales, even if they are only identified up to the genus/family rank. This contrasts with most currently available databases (including ours), where a few conspicuous taxa are overrepresented.

According to the relationship between the position each species occupies within an ecological network and its ecological “importance” (understood as the degree of ecosystem structural alteration that would result from its extinction) [11,19,45], modularity analyses stressed that “bridge” species linking ecological compartments and species occupying within-module hubs may disproportionately contribute to network identity [44]. Rarefaction experiments further indicate that such key organisms tend to be medium-sized animals occupying intermediate trophic levels. Moreover, multilayer networks underscored the importance of indirect developers and organisms with amphibious lifestyles in functionally integrating heterogeneous habitats. These organisms are therefore critical for maintaining ecosystem stability and should thus become the primary targets of conservation efforts [11,46].

However, some of the identified functional or life-history traits of key species (e.g., the prominence of amphibious organisms) are likely specific to wetland ecosystems, and conservation strategies for other ecosystem types therefore warrant further investigation. Nevertheless, our automated, data-driven framework is also well suited for application across a broader range of ecosystems. This is partly because many ecosystems are structurally simpler than wetlands (in terms of sub-habitat complexity rather than species richness), and partly because several of the functional traits identified by the algorithm—such as intermediate trophic position, trophic generality, and high mobility—are broadly relevant determinants of species’ ecological roles across systems. As such, the method provides a transferable tool for identifying ecologically important species beyond wetlands, while still allowing ecosystem-specific traits to modulate the outcomes. Finally, our modularity analysis emphasizes that protecting key species (rather than ecosystems as a whole) may only be more effective in small systems, as the number of critical “bridge” species increases with species richness.

Another notable finding refers to the relevance of temporal scale and the concept of species *potentiality*, i.e., the distinction between a species’ full ecological potential and its actual interactions within a given spatiotemporal context. This distinction appears not only crucial for understanding eco-evolutionary dynamics [47] but, as our results indicate, for interpreting and comparing network structure. In many published food-web networks, generalist species appear as densely connected *hubs* because they integrate all possible diets recorded over years [48]. However, real selective pressures are mostly shaped by local, individually-experienced conditions, such as phenological availability of preys or stochastic encounters, rather than by all the full range of conditions that can be *potentially* experienced at species-level [39]. Our approach aligns with this perspective by showing, in each replicate, only the realized interactions of species within a given timeframe. This avoids the artefactually high connectivity of generalists introduced by classic approaches and argues against defining key species solely based on their trophic niche amplitude [45].

This method is likely to be refined and improved; for instance, incorporating non-trophic (e.g., pollinator-plant) and non-binary interactions [32,45,49,50], accounting for the biomass/population sizes of organisms to model ecological dynamics [36,51,52], or integrating new sources of functional information (e.g., tooth complexity or isotopic analysis of the enamel) to better resolve the plant-herbivore interactions [53]. Although these upgrades represent a big challenge (especially for multilayer extensions), the general idea of algorithmic data-mining and processing (e.g., [52]) and automatic model construction may still apply [5].

Even with the minimal theoretical ecology principles currently implemented, our procedure enables the rapid characterization of ecosystem structure, functional modules, and key species. This efficiency extends to multilayer networks, which have largely been limited to theoretical investigations due to the immense sampling effort required to record all ecologically relevant aspects (developmental modes, seasonality, etc.) of organisms. We argue that the presented approach not only deepens our understanding of ecosystems (both contemporary and extinct), but can also potentially serve as a valuable tool to promote more rational strategies to habitat and species conservation, an urgent need in the context of the ongoing biodiversity crisis.

## Materials and methods

Our aim is to construct realistic trophic networks directly from raw, non-curated species lists, a type of information available for many extant and extinct ecosystems. The proposed protocol consists of three main stages: (i) data acquisition and preprocessing of species lists, (ii) automatic enrichment of these lists with key functional traits mined from public biodiversity databases, and (iii) automatic generation of trophic networks using a custom-made algorithm based on the allometric niche-model framework. The following sections describe these procedures and, additionally, the analyses performed to validate our method.

### Protocol for data collection and processing

To assess the validity and generality of our approach, we deliberately focused on ecosystems exhibiting substantial structural complexity, allowing the method to be evaluated under realistic and heterogeneous conditions. Wetlands and wetland-like ecosystems—including estuarine habitats, small lakes, and riverbanks—provide an ideal testbed in this regard, as they are characterized by pronounced spatial compartmentalization across water–land–air interfaces, strong temporal dynamics (e.g., seasonality and tides), and high levels of biodiversity [28]. Accordingly, the dataset used in this work comes from the RAMSAR wetland database (https://rsis.ramsar.org/), which contains ≈2500 protected wetland-like ecosystems worldwide. To maximize habitat heterogeneity, we divided their global distribution into 18 biogeographical regions, each containing a similar number of RAMSAR sites (panel 1 in Figs 1 and SI). Within these regions, we manually extracted all species lists containing >30 species and at least 10 primary producers. The resulting dataset comprises 160 sites encompassing >27000 taxa (N_max_ = 2181 species). This sample size exceeds that of most comparative analyses of ecosystem-level networks [6,7,29,30,32,54]; a feature that allows us to demonstrate the scalability of the method. Importantly, species lists in this database are neither standardized nor taxonomically homogeneous and often contain invalid or ambiguous names, which allowed us to test the robustness of our algorithm against different sources of noise (see below).

### Automatic data inflation

Although trophic interactions involve many types of traits (e.g., venom/resistance, location/avoidance, etc.) [54,55], a few key functional traits such as body size can successfully predict the trophic position of species [31,54,56,57]. Accordingly, the raw species lists were supplemented with body size, body mass, diet, and habitat attributes. To automatize the process, we developed custom-made search algorithms applied to two large, open-access biodiversity databases: the 2021 “*Catalogue of Life*” (https://catalogueoflife.org/, hereafter COL, ≈ 2 × 10^6^ species, [48]); and the 2023 “all trait dataset” from the *Encyclopedia of Life* (https://opendata.eol.org/, hereafter EOL, ≈ 2.4 × 10^6^ species and ≈19.2 × 10^6^ traits). Importantly, such algorithms incorporate mechanisms to correct erroneous data and protocols to infer the missing data, either from previously known body mass/size relationships or from phylogenetic inference (see S1 Text and Table A in S1 Text for details).

### Automatic network construction

Plausible trophic networks were automatically constructed using a version of the Allometric Niche Model (ANM) [14], which assumes that the trophic position of a species (0 < *n*_*i*_ < 1) strongly depends on its relative (log) body mass among ecosystem species [22,45,46]. Besides *n*_*i*_, each species “*i*” in the ANM has a feeding optimum *c*_*i*_ and a feeding range *r*_*i*_. Therefore, a species *i* will predate on every species *j* whose trophic position *n*_*j*_ lies within the interval [*c*_*i*_*-r*_*i*_*/2, c*_*i*_ *+ r*_*i*_*/2*] (see panel 6, Fig 1). Following the ANM, the feeding optimum (*c*_*i*_) was drawn from a uniform U~[*r*_*i*_*/2, n*_*i*_] distribution, and the feeding range (*r*_*i*_) from a Beta~[*α = 1, β = 2C*] distribution, where C denotes network connectivity (estimated from empirical data, see Table C in S1 Text). Primary producers can be potentially consumed with a probability C by any herbivore irrespective of its size (as herbivores do not generally consume the whole plant, the plant-herbivore allometric relationship vanishes, [37]). Although more accurate ways to discriminate plant-herbivore interactions can be envisioned (see Discussion), both statistical analysis (S1 Fig) and rarefaction experiments (Fig 8A-8D) suggest that our agnostic approach suffices for a preliminary incorporation of primary producers to the interaction network (as it does not introduce any consistent, directional bias).

After establishing the possibility of a trophic relationship between two species based on their relative sizes (ANM criterion), we applied a second criterion considering the trophic preferences of the larger species and the taxonomic category of the smaller species. For instance, if “A” is an insect and “B” is an insectivore animal, then the trophic interaction A → B is generated (proven the ANM criterion has been previously fulfilled for that interaction). To avoid spurious interactions, a categorical coarse-graining filter was applied to the dietary attributes (see SI).

When all potential trophic interactions were assessed, we checked whether every animal in the resulting network feeds on something (beyond cannibalism), and every producer is eaten by at least one animal. If spare nodes appeared (e.g., due to uneven sampling, some species lists include obligate insectivores but no insects), we relaxed the interaction criteria to force the generation of a fully connected network (see SI). The outcome of this multi-step algorithm is a set of binary trophic networks (one per replicate) for each ecosystem, plus an additional file containing the complete taxonomic and functional information of all taxa. For the i^th^ network, the element T_i_(*j,k*)=1 if the species *j* consumes the species *k* and zero otherwise (links denote trophic interactions and not merely species co-occurrence). To manage the exponential increase in computational effort with species richness (N), we made the number of replicated networks inversely proportional to log(N). No population sizes or biomass transfers were considered. Contrary to other procedures [29,30,33], redundant nodes (i.e., functionally analogous species) were conserved, as their removal may alter some descriptors which are relevant to our comparative studies. All output files are generated in *.csv format, enabling them to be easily shared and analysed by widely used software among ecologists (e.g., *Gephi, Cheddar*) [6].

### Multilayer networks

To construct multilayer networks considering habitat compartmentalization and life stages, we extracted from the EOL database the habitat of organisms: if only “terrestrial” entries were found (e.g., forest), both adult and larval stages of that organism were placed in the terrestrial layer; whereas if only “aquatic” entries were found, both stages were assumed to be aquatic. For organisms showing mixed attributes, we applied a phylogeny-based criterion to assign habitats and diets to adults and larvae, which is supported by the fact that life-history traits are, up to certain taxonomic levels, evolutionary conserved [36]. A similar, phylogeny-based criterion was used to represent the ability of organisms to trespass the air-water interface once in their lifetimes (e.g., mosquitoes) or several times (e.g., frogs). Upon that multilayer structure, the ANM was applied, but taking into account two extra considerations: (i) that the body size of the larvae was reduced by one order of magnitude with respect to the adult, which is a reasonable approximation for many taxa [29], and (ii) that a trophic interaction only appears if, in addition to the allometric (body size) and the dietary-preference criteria, the two organisms share the same abstract region within the multilayer network [2]. These rules were codified in a matrix operator, which defined a 2-layers × 2-coordinates architecture. Large (N > 200 species) ecosystems were discarded, as the potential multilayer network would be too big (>10^5^ links).

### Network analysis

For each replicated network, we calculated a set of standardized topological descriptors routinely used in theoretical ecology (Box 1) [14,29,30,33,57]. These descriptors evaluate the biological realism and structure of the networks, their resemblance to empirically derived networks, and their resilience against diverse sources of perturbations in the data structure. For instance, in the (random) rarefaction experiments, different proportions of the species (0, 0.25, 0.5) were randomly removed from each ecosystem species list. From those perturbed lists, new (perturbed) networks were generated, and their structural properties compared with those of the unperturbed network. The detailed analysis of specific wetland sites and taxa is beyond the scope of this methodological paper and will be presented in a follow-up publication.

Box 1 Quantitative descriptors of the networks used for analytic and comparative purposes. This box provides a consolidated reference for all topological and non-topological metrics, including their acronym and definition, to facilitate quick access without searching through the main text. Topological descriptors were measured over the (binary) trophic matrix T (N × N), non-topological descriptors are marked with *, and (topological) vectorial magnitudes are underlined. Some of these descriptors capture overlapping aspects of network structure and are thus not statistically independent.

• Species richness (N*), number of genera (G*), families (F*), orders (O*), classes (CL*), phyla/divisions (P*), average body size and body mass (ABS* and ABM*, primary producers excluded).
• The diversity index β* (based on [36]) measures how “unique” is the species composition of each ecosystem respect the whole set of wetlands. β is calculated as a Jaccard index: βi = 2C/(N_i_ + N_T_), being C the number of common species between the i^th^ ecosystem and the whole species lists of all ecosystems (except itself); N_i_ the species richness of the i^th^ ecosystem and N_T_ the whole number of species. If β = 1, the ecosystem does not contain exclusive species, whereas if β = 0, all its species are unique. We calculated β for all taxonomic ranks.
• **Average number of links** (interactions) per species, **AVL** = ∑(T)/N; and directed connectivity CON = ∑(T)/N^2^.
• **Average in-degree (IND)**: How many species are eaten, on average, by a given species. **Average out-degree (OUT)**. How many species, on average, fed on a given species. The **IND/OUT asymmetry**, relevant for the stability of the community [50], is denoted as ASYM.
• Degree distribution (DEG). Recorded in the vector DEG, where the ith element represents the relative proportion of species having exactly “i” trophic links (i.e., that eats or is eaten by “i” species). **Generality (GEN)** and **Vulnerability (VUL)** represent, respectively, the degree distributions resulting from counting only the (in-degree) links from resources to consumers; and from consumers to resources (out-degree) [25,47].
**• MAXL, MAXIND** and **MAXOUT:** Respectively, the maximum number of species that can interact with, be eaten by, or fed on a single species. **TOP, BAS**: The fraction of species that are top predators (not eaten by any other species) or basal (primary producers and detritivores). **INT**(=1-TOP-BAS): % of species that occupy intermediate trophic levels. We also calculated the producers/consumers ratio **PC** = BAS/(1-BAS), commonly used to assess the long-term sustainability of economically relevant communities [36].
**• AVGL, MINL** and **MAXL**: Average, maximum and minimum trophic (Cohen) chain lengths. That is, the average, maximum and minimum number of possible trophic levels [58].
• **NCHA**: Number of different trophic (Cohen) chains. Two trophic chains are considered as different if at least one of their non-zero elements are different. For instance, the chains {S1 → S2 → S3} and {S1 → S2 → S4} are different, whereas the chains {S1 → S2 → S3} and {S1 → S2 → S3 → S4} are not.
• **NLOOPS**: Number of closed loops. Loops can appear when species occupy similar trophic levels, and when there are considerable differences between the adult and juvenile body sizes, so that the adult of the species A can feed on the larvae/juveniles of the species B, and vice versa [58]. The number of loops of length = 1 is also recorded, and calculated as **LOOP1** (=|T-T^T^|/(2N^2^), where T^T^ is the transpose matrix).
• **Mean modularity (MOD)** The degree to which a network can be partitioned into distinct groups of nodes (i.e., modules, communities or partitions) with denser internal connections compared to inter-module connections. A high modularity denotes the existence of well-defined trophic modules whose species tend to interact with themselves. We also quantify, for each ecosystem, the number of partitions in which it can be divided (**PART**), and its average clustering coefficient (**CLUST**): the proportion of links between every node and its neighborhood divided by the total number of links that could possibly exist between them.
• **Nestedness (NEST)** In biological systems (especially in those formalizable as bi-partite networks), nestedness describes how prone are the specialized organisms of the type-A (e.g., pollinators) to interact with generalist species of the type-B organisms (e.g., plants), and vice versa [32]. Yet different metrics have been proposed, we adopt here, for its ease of implementation and widespread use [32,49] a distance-based approach that quantifies the deviation (% of misplaced links) of the network taking as a reference a fully nested network of the same size [59].

## Supporting information

S1 Fig Some topological properties remain relatively insensitive to variations in the % of primary producers (BAS, indicated on the X-axis in all panels). Top row (L to R): The number of species (N) does not escalate with BAS, which suggests that variation in BAS corresponds to a biological reality rather than to a systematic sampling bias. BAS is relatively independent of the number of top predators (TOP). Obviously, some statistical descriptors will be affected by BAS, such as the % of intermediate nodes INT (defined as 1-TOP-BAS). Middle row (L to R): While the average number of links (AVL) and connectivity (CON) are mildly affected by BAS, the average in-degree (IND) is strongly affected, as it measures how many species are eaten, on average, by a given species (i.e., resource-consumers flows). Bottom row (L to R): Most of the descriptors related to the length or number of (Cohen) trophic chains (MAXL, NCHA), or to the quantitative differences with pure niche models (denoted by the “M” exponent in DEG) vary little with BAS.
(TIFF)

S2 Fig Effect of data quality in network structure. In these plots, a percentage of the original input data (horizontal axes) is substituted by random or arbitrary values. Vertical axes represent how different are the networks resulting from randomized data compared to the original networks (i.e., the ones using the whole functional information about species). Differences with the original networks are plotted as relative values, using the standard deviation among replicates (of the original networks) as a range of confidence (dotted lines). Each point corresponds to a (randomized) network replicate of a given ecosystem, and colors denote which type of topological attributte is considered (see color code in A). If most topological attributes of the randomized networks fall within those dotted lines, the data quality has a mild effect on the ability of our algorithm to reconstruct realistic trophic networks. This is the case for randomization of the dietary preferences of the species (panels A, B). However, when dietary preferences are substituted, even in a small proportion, by a fixed value (e.g., herbivory), topological attributes are distorted (especially those related with overall connectivity such as AVL, CON, IND, etc., see main text and panels C, D). The similarity between A, B and B-D panels suggests that, compared with dietary preferences, randomization of size attributes (e.g., body mass) changes little the network structure (as missing or erroneous data are, first, corrected by the search algorithm itself and then, compensated by the ANM, see Methods). These findings help understanding trophic network self-organization: while relative body sizes primarily determine the network topology, the finer functional attributes, such as dietary preferences, play a key role in arranging species within that structure.
(TIFF)

S1 Text Supplementary Information of the paper. (PDF)
