# Supplementary figures and images for: Algorithmic reconstruction of trophic networks from open-access species lists reveals key organisms in real ecosystems
Source: PLoS Comput Biol. 2026 Mar 12;22(3):e1014061. doi: 10.1371/journal.pcbi.1014061 (PMC13001971; doi:10.1371/journal.pcbi.1014061)

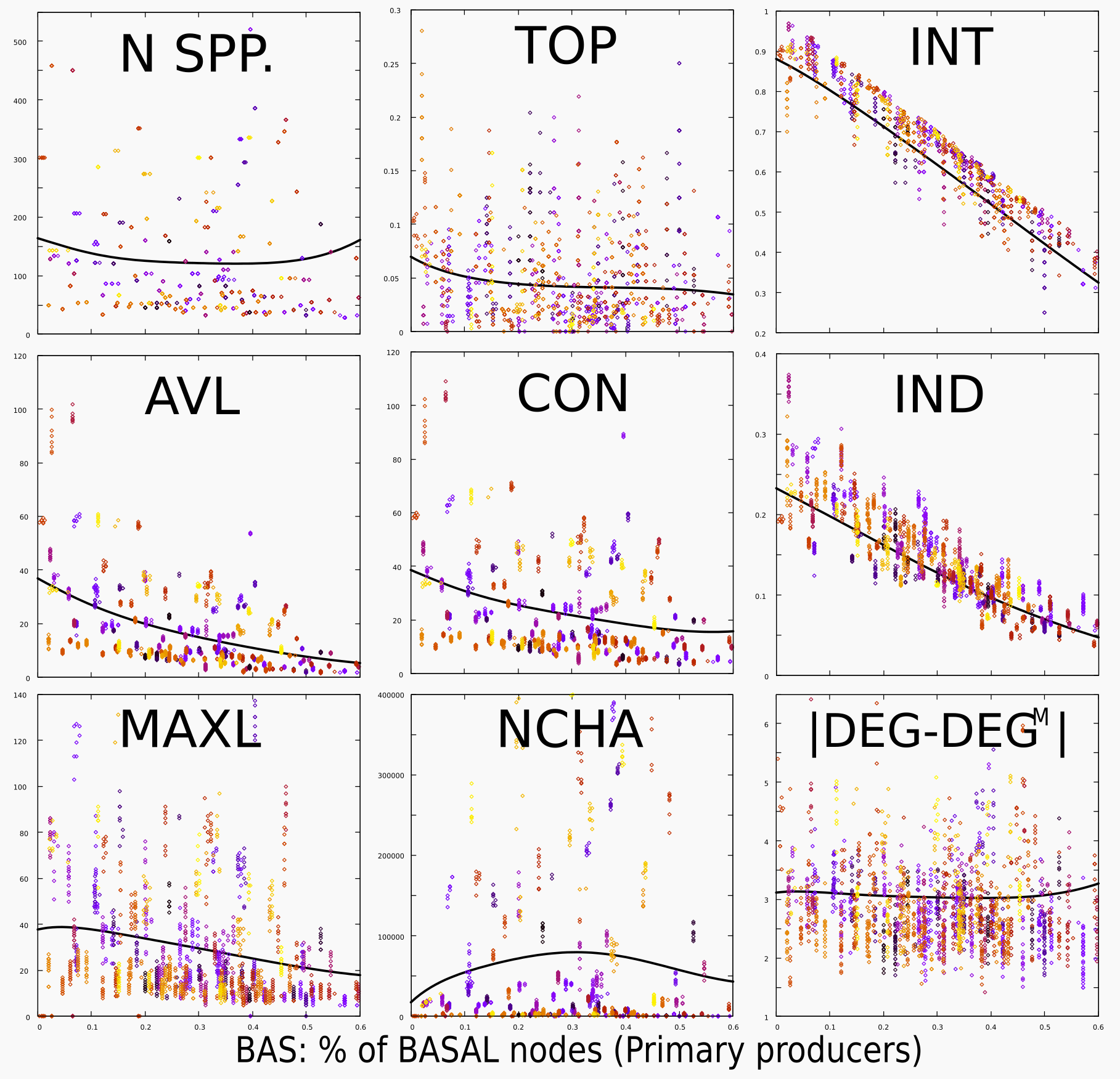

Supplement: S1 Fig — Top row (L to R): The number of species (N) does not escalate with BAS, which suggests that variation in BAS corresponds to a biological reality rather than to a systematic sampling bias. BAS is relatively independent of the number of top predators (TOP). Obviously, some statistical descriptors will be affected by BAS, such as the % of intermediate nodes INT (defined as 1-TOP-BAS). Middle row (L to R): While the average number of links (AVL) and connectivity (CON) are mildly affected by BAS, the average in-degree (IND) is strongly affected, as it measures how many species are eaten, on average, by a given species (i.e., resource-consumers flows). Bottom row (L to R): Most of the descriptors related to the length or number of (Cohen) trophic chains (MAXL, NCHA), or to the quantitative differences with pure niche models (denoted by the “M” exponent in DEG) vary little with BAS. (TIFF) [file pcbi.1014061.s001.tiff]

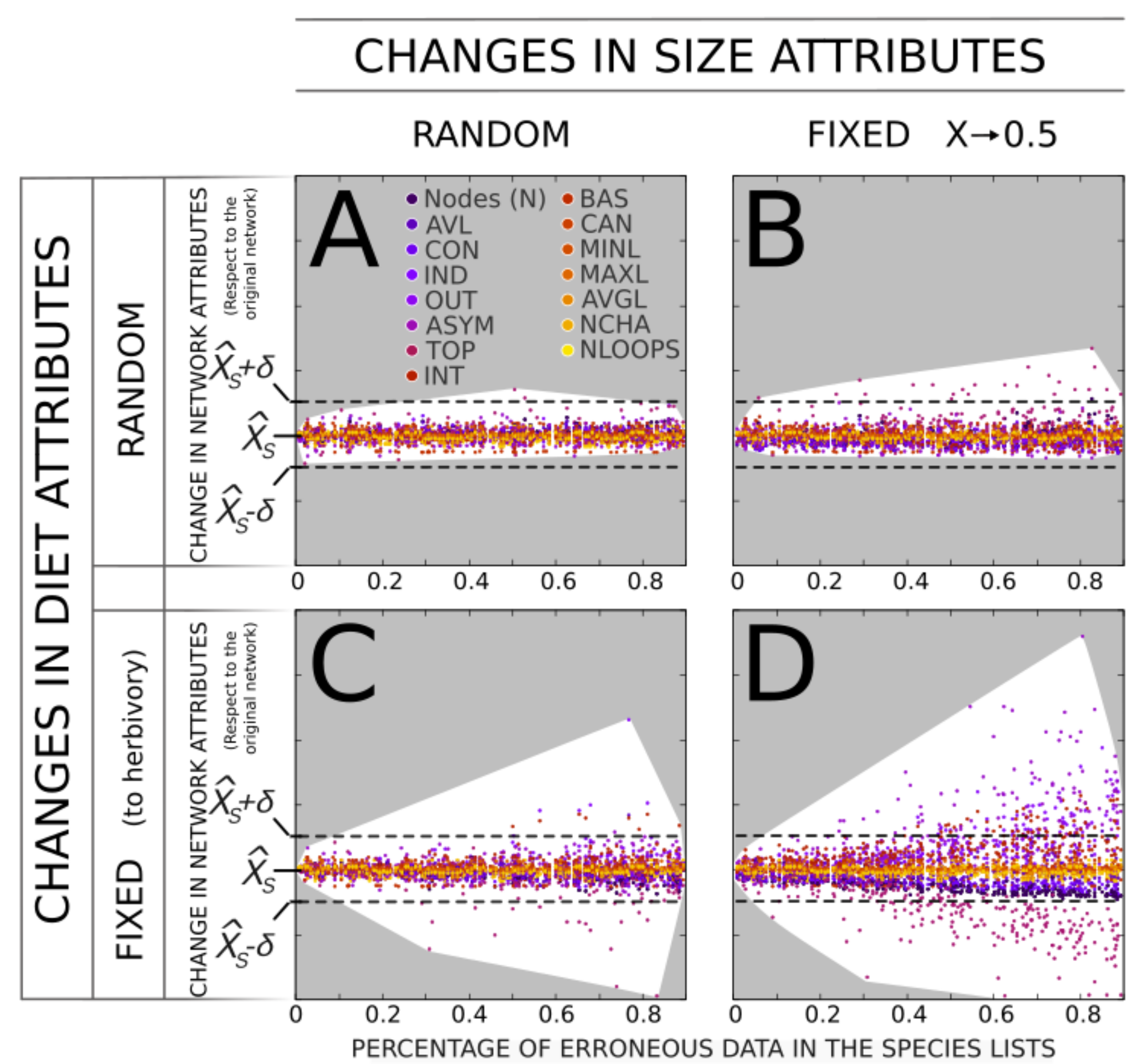

Supplement: S2 Fig — In these plots, a percentage of the original input data (horizontal axes) is substituted by random or arbitrary values. Vertical axes represent how different are the networks resulting from randomized data compared to the original networks (i.e., the ones using the whole functional information about species). Differences with the original networks are plotted as relative values, using the standard deviation among replicates (of the original networks) as a range of confidence (dotted lines). Each point corresponds to a (randomized) network replicate of a given ecosystem, and colors denote which type of topological attributte is considered (see color code in A). If most topological attributes of the randomized networks fall within those dotted lines, the data quality has a mild effect on the ability of our algorithm to reconstruct realistic trophic networks. This is the case for randomization of the dietary preferences of the species (panels A, B). However, when dietary preferences are substituted, even in a small proportion, by a fixed value (e.g., herbivory), topological attributes are distorted (especially those related with overall connectivity such as AVL, CON, IND, etc., see main text and panels C, D). The similarity between A, B and B-D panels suggests that, compared with dietary preferences, randomization of size attributes (e.g., body mass) changes little the network structure (as missing or erroneous data are, first, corrected by the search algorithm itself and then, compensated by the ANM, see Methods). These findings help understanding trophic network self-organization: while relative body sizes primarily determine the network topology, the finer functional attributes, such as dietary preferences, play a key role in arranging species within that structure. (TIFF) [file pcbi.1014061.s002.tiff]
