# Supplementary material for: Algorithmic reconstruction of trophic networks from open-access species lists reveals key organisms in real ecosystems
Source: PLoS Comput Biol. 2026 Mar 12;22(3):e1014061. doi: 10.1371/journal.pcbi.1014061 (PMC13001971; doi:10.1371/journal.pcbi.1014061)
Supplement: S1 Text — (PDF) [file pcbi.1014061.s003.pdf]

Supplementary Information of the paper:

Algorithmic Reconstruction of Trophic Networks from Open-Access Species Lists Reveals Key Organisms in Real Ecosystems

Miguel Brun-Usan[1,*], Roberto Latorre[2], Ángela D. Buscalioni[1], Paloma Alcorlo[3,4], Jesús Marugán-Lobón[1]

1- Dpto. Paleobiología / CIPb (Centre for the integration of Paleobiogy). Universidad Autónoma de Madrid (UAM), Madrid, Spain.
2- Dpto. Ingeniería Informática. Escuela Politécnica Superior. Universidad Autónoma de Madrid (UAM), Madrid, Spain.
3- Dpto. Ecología. Facultad de Ciencias. Universidad Autónoma de Madrid (UAM), Madrid, Spain.
4- Centro de Investigación en Biodiversidad y Cambio Global (CIBC). Universidad Autónoma de Madrid (UAM), Madrid, Spain.
*m.brunusan@gmail.com

Supplementary methods:

Biogeographical sectorization of RAMSAR sites:

Neotropical and Afrotropical regions were sectorized along the tropics and equatorial lines, while Palearctic region was divided using the 90ºE and 45ºE meridians. Europe (the richest region in RAMSAR sites), was isolated from the rest of Palearctic region and subdivided in four quadrants to distribute more evenly the sampling effort. India and Madagascar, two biodiversity hotspots, were considered separately. Australia and the Polynesian islands were separated from SE Asia by the Wallace line.

Automatic data inflation protocol:

The automatic data inflation consisted of the following steps:

1. *Automatic taxonomic classification*. For each entry in a raw species list, a full taxonomic classification was recorded, including all possible synonyms of the entry to make the search of functional traits more exhaustive. Taxonomic information was first searched in COL (as it keeps very comprehensive lists of synonyms), redirecting the search to EOL if no reliable classification was found. Subspecies were collapsed into the species-level.
2. *Manual correction*. Due to the use of synonymous, homonymous, vernacular, misspelled, or obsolescent names, ≈3% species in the input lists were not listed neither in COL nor in EOL. These cases were manually corrected after surveying specialized literature and the Global Biodiversity Information Facility (www.gbif.org), looking at the original RAMSAR lists to select the appropriate entry, and then re-launching the automatic taxonomic classification.

3. *Automatic search of traits*. Given a list of synonyms, we conducted a search in EOL to identify the corresponding functional traits. Body size and body mass attributes were computed as maximum numerical values (to avoid distorted average values due to repetitive data). In the realm of plants, sizes and weight data exhibited significant heterogeneity, often referring to different structures (e.g., leaves, fruits, seeds). To keep consistency with animal data, and considering that the size/mass of primary producers was not crucial for estimating the network structure (given our assumption that they are potentially consumable by any herbivore regardless of their size), plant sizes and masses were uniformly set to zero.

   Diet and habitat were incorporated into the extended dataset as multi-evaluated attributes. The absence of a standardized semantic framework within EOL for uniformly designating all possible attributes and their values introduced complexities in the search process. On one hand, it led to variations in the nomenclature of attributes, where different attributes might refer to the same trait (e.g., "diet", "diet breadth" or "diet includes" for the diet of a species). On the other hand, in qualitative attributes, it resulted in the possibility of values at different levels of granularity (e.g., "carnivorous", "insectivorous", "insects" or "spiders" were valid values for the diet trait). To address attribute synonyms and guide data acquisition, we implemented a coarse-graining filter (see next section).

4. *Automatic data completion.* Unfortunately, after the automatic search of traits, ≈7% of species still lacked part of the required data. Those cases were handled as follows:

   a) Missing body size or body mass were inferred based on well-known taxon-specific allometric correlations from literature (See Table A below). In this table, the ***a*** and ***b*** parameters correspond to taxon-specific scaling (i.e., allometric) coefficients. These parameters, recorded from the literature (references provided), allow us to infer the missing data for species presenting a bodysize but not a bodymass, or vice versa. The column "model" corresponds to the type of mathematical function used, namely:

      1. Linear: $W=a+b(L)$
      2. Logarithmic: $log(W)=a+b\cdot log(L)$
      3. Power: $ln(W)=a+b\cdot ln(L)$
      4. Exponential: $ln(W)=a+b\cdot(L)$

      Where W is bodyweight and L is bodysize, both transformed into SI units (grams, mm.). If different studies use different models for the same taxon, we choose the one with a better fit (third column in Table A). If more than one study had addressed the mass-bodysize relationship of the same taxa using the same model but different samples

(and thus providing slightly different scaling coefficients), we chose the coefficients of the study containing a larger sample (fourth column in Table A).

For some taxa, the recorded bodysize did not correspond to the animal length, but to some specific standardized measures (e.g., major shell axis for bivalves, cephalon/thorax and cephalothorax width for some crustaceans, SVL, etc.). In those cases, we checked that the measure provided was consistent with the measure that was used to determine the allometric scaling.

b) For entries where both body size and body mass were lacking, and/or with no diet or habitat, a taxonomic ladder-climbing algorithm was applied. Thus, if a trait was not found for a given species, the algorithm looked for the value of the missing trait in all the species of the genus to which that species belongs. Similarly, when a trait was missing for an entire genus, the search was extended to the whole corresponding family, and so on, until the automatic completion of all required data.

Taxa	*a*	*b*	*Model*	*Ref.*
Annelida (polychaeta)	-4	2.5	2	[1]
Ascidiacea	-3.2	2.1	2	[1]
Echinodermata	-3	2.5	2	[1]
Gastropoda	-3.6	2.6	2	[1]
Pellecipoda (longest axis)	-4.2	2.8	2	[1]
Arachnida	-3.3	2.9	3	[2]
Chilopoda	-4.1	2.2	3	[2]
Crust. Decapoda (width)	-3.5	3.1	2	[1]
Crust. Amphipoda	-3.4	2.3	2	[1]
Crust. Isopoda	-4	1.9	2	[1]
Insecta(all)	-3.5	2.4	3	[2]
Diptera	-3.6	2.5	3	[2]
Hymenoptera	-3.3	2.1	3	[2]
Formicidae	-3.9	2.5	3	[2]
Coleoptera adult	-3.3	2.7	3	[2]
Lepidoptera adult	-4.8	2.8	3	[3]
Coleoptera larvae	-7.1	0.8	1	[3]
Lepidoptera larvae	-0.6	0.1	4	[3]
Orthoptera	-3.5	2.4	3	[3]
Hemi/homopterans	-3.3	2.4	3	[3]
Collembola	-1.9	2.3	3	[3]
Fishes (all)	-4.65	2.95	2	[1]
Gadiformes	-4.9	2.9	2	[1]
Gobiesociformes	-4.6	2.7	2	[1]

Taxa	*a*	*b*	*Model*	*Ref.*
Perciformes	-4.9	3.1	2	[1]
Pleuronectiformes	-5.2	3.1	2	[1]
Anura	-4.33	3.16	3	[4]
Caudata (SVL)	-4.15	2.6	3	[4]
Agamidae (SVL)	-4.774	3.073	1	[5]
Chamaeleonidae (SVL)	-3.997	2.68	1	[5]
Gekkonidae (SVL)	-4.495	2.9	1	[5]
Iguanidae (SVL)	-4.298	2.972	1	[5]
Lacertidae (SVL)	-4.543	2.951	1	[5]
Scincidae (SVL)	-4.821	3.029	1	[5]
Teiidae (SVL)	-4.747	3.11	1	[5]
Varanidae (SVL)	-5.301	3.235	1	[5]
Sauria (SVL)	-4.717	3.027	1	[5]
Anguimorpha (SVL)	-5.058	3.145	1	[5]
Gekkota (SVL)	-4.495	2.9	1	[5]
Iguania (SVL)	-5.033	3.243	1	[5]
Scincimorpha (SVL)	-5.148	3.244	1	[5]
Mammals (all)	0.359	2.501	1	[6]
Marine mammals	0.34	2.587	1	[6]
Wholly-marine mammals	0.316	2.717	1	[6]
Terrestrial mammals	0.357	2.498	1	[6]
Volant mammals	0.317	2.369	1	[6]
Non-volant mammals	0.334	2518	1	[6]

Table A. Taxon-specific parameters for the allometric scaling. ***a*** and ***b***: scaling coefficients. SVL: Snout to vent (cloaca) length. See references below.

Coarse-graining filtering and algorithmic treatment of the minority trophic categories:

To compensate for the absence of a common semantic framework in EOL, our coarse-graining filter assigned the value "herbivore" to whichever of the expressions {seed, leaves, grass, tree, lichen, algae, fruit, wood, bark, weed, pollen}. Likewise, non-biological categories such as "worm" or "fish" were assigned to the appropriate taxa (see Table B below). When multiple diets were present for a given species (e.g., {plant, carnivore, arthropod, insect}), the algorithm distributed probabilities to each category proportional to the number of tokens relative to each category and level of granularity. For instance, in the previous example, the species will behave as a carnivore on 75% of the time, and as an herbivore 25% of the time. Additionally, more detailed feeding preferences (e.g., insectivore) were established, if present, for carnivores, slightly favoring trophic specialization across replicates. That is because, in large ($N \geq 50$) communities, the number of specialists tends to outnumber that of generalists to minimize the overlap between ecological niches (see S1 Text references [7,8] below, and ref. [53] in the main text).

Some minority trophic categories required more detailed consideration. For example, the categories {detritivore, sedimentivore, suspended-matter, MOR (=humus)} were treated as primary producers capable of growth by using only abiotic nutrients. The alternative strategy (creating an *ad hoc* "detritus" node and treating it as a species as in (see S1 Text references [9,10]), was discarded because such a node should then have as many inputs as species and as many outputs as detritivores, resulting in an artefactual hub that could significantly distort the network topology. Scavengers were categorized as carnivores with no allometric relationship with their preys, since they can consume species larger than themselves. Ecological strategies involving cooperation to capture larger preys (e.g., wolves, eusocial insects, etc.) were not considered. However, since many social hunters are also scavengers, they were still adequately treated by the algorithm.

Finally, for (animal) parasites, the "inverse niche model" was applied (see S1 Text reference [11]). According to this model, the trophic optimum of a parasite is "above" its body size (drawn from U~$[nr, 1\text{-}rr/2]$), and its feeding range *decreases* with its trophic position, as $rp=(1-np)yp$, where yp Beta~$[\alpha=1, \beta]$. Plants labeled as parasites (e.g., mistletoe, *Viscum album*) were treated as herbivores (only concerning their trophic preference, not their taxonomical category) as their hosts are always other plants. Likewise, plants labeled as {carnivore, insectivore, insect, spider, ...} were assigned an additional trophic category, carnivore/insectivore, alongside their by-default classification as primary producers. When present, the niche parameters (n_i, c_i and r_i) for these carnivorous plants were set equal to the minimum value found for animals in that ecosystem ± their respective square roots. These general rules, derived from fundamental principles in biology and logic, enabled our protocol to automatically handle highly specific interactions (see Fig.2 in main text).

Input data		Final Category	Input data	Final Category
autotroph	endosymbiont	Autotroph	alga	Herbivore
chemoautotroph	phtosynthesis		bark	
chemotroph	symbiotroph		branch	
acari	larvae	Carnivore	cellulose	
ant	lizard		fern	
antelope	microinvertebrate		flower	
apexpredator	millipede		foliage	
aphid	mollusc		fruit	
arthropod	nematode		fungi	
ascidia	nestling		grass	
bee	newt		herbivore	
beetle	octopus		leaf	
bird	ostracod		leaves	
butterfly	polip		lichen	
carnivore	predator		microalgae	
caterpillar	reptile		moss	
centipede	scavenger		nectar	
cephalopod	seacucumber		nectarium	
collembola	seal		phytoplankton	
copepod	seasquirt		plant	
coral	seastar		plantissue	
crab	seaurchin		pollen	
cricket	shrimp		root	
crustacea	slug		sap	
cub	shell		seagrass	
cuttlefish	snail		seaweed	
dragonfly	snake		seed	
earthworm	spider		weed	
echinoderm	sponge		wood	
eel	springtail		blood	Parasite
fish	squid		ectoparasite	
frog	tadpole		egg	
grasshopper	termite		endoparasite	
honey	vertebrate		feather	
insect	wasp		hair	
invertebrate	waterflea		hemolymph	
jellyfish	worm		parasite	
krill	zooplankton	Scavenger	scale	
carrion	deadanimals		skin	
corpse	scavenger		tissue	
bacteria	litter	Detriti-Vore	omnivore	Omnivore
clay	microorganisms		plankton	
decayingmatter	MOR			
detritivore	organicmatter			
detritus	organicparticles			
filterfeeder	protozoa			
hyphae	sediment			
leaflitter	suspendedmatter			

Table B: Input→output relationships used in the taxonomic coarse-graining filter. In this table, the columns "input data" contain the character strings (without spaces) as found in databases, and the output columns represent the final trophic category assigned to each taxon. Organisms belonging to each trophic category are treated differently when the network is constructed: **Autotrophs** (except insect-eater plants) lack input edges, but they have to have at least one organism that feeds on them (i.e. they have to have at least one output edge). **Carnivores** must have at least one animal as input node. The ability of carnivores to feed on other animals is determined by their relative bodysize, as established by the allometric niche model. The model also considers, to some extent, trophic specialization within this general category (e.g., insectivore, see above). **Scavengers** are treated as carnivores, but without the allometric restriction (scavengers typically

feed on dead animals larger than themselves). **Detritivores**, as autotrophs, lack input edges, and they also have to have at least one organism that feeds on them (i.e., they have to have at least one output edge). **Herbivores** must be connected to at least one autotroph input node (the allometric niche model does not apply). This category includes animals that are said to “parasitize” plants, such as aphids. **Parasites**. If the focal organism is a plant, it is assumed to parasitize other plants, and thus treated as a herbivore. If the parasite is an animal (and parasites other animals), then the inverse niche model applies (see above). **Omnivores**. If only this label is found, the organism is treated as a carnivore, or as a herbivore, with a 50%-50% probability. These categories are not mutually exclusive. When organisms belong to more than one trophic category, the relative number of assignations to each category is taken as a proxy for its trophic preferences (see above).

Estimation of connectivity C:

In our approach, the network connectivity C (the number of realized trophic links (L) among all the possible links between the S species, $C=L/S^2$) is a parameter that needs to be necessarily introduced in the allometric niche model to calculate the feeding range (r_i) of each species. As C is unknown prior to the simulation of the network, we assign to each replicate a C drawn from a N~($\hat{C}$,σ_C) distribution with mean ($\hat{C}\approx0.12$) and variance ($\sigma_C\approx0.05$), both estimated from empirical data (see Table C below). In this table, the “Taxa” column represents the number of different taxa in the considered ecosystem, while S represents the number of functionally distinct nodes. The value of S is always lower or equal than “Taxa”, as two closely related species might occupy an identical trophic niche (at least within the resolution level of the study, see main text for an appraisal on real niche dimensionality). The column L represents the number of realized edges in that network, that is, the number of empirically established trophic interactions between the S species. The L/S column represents the average link density, and C (L/S^2) represents the network connectivity, which is the proportion of all the possible trophic interactions (S^2) that is actually realized. The bottom row contains the average values of each topological descriptor. The average value of C ($\hat{C}\approx0.12$) is used as an input parameter to calibrate the allometric niche model (using in each replicate a C drawn from a N~(0.12, 0.05) distribution. In some simulations that start with very low C values, the resulting networks eventually show disconnected nodes. If this happens, the network connectivity is progressively increased to favour the connection of the spare nodes with other organisms. In these cases, the maximum recorded value of C ($C_{MAX}\approx0.41$, corresponding to a marine ecosystem) allows us to define a maximum threshold for C. If that C_{MAX} limit is reached, the spare node is randomly connected (see Methods).

Ecosystem	Taxa	S	L	L/S	C(L/S²)	Ref.
Benguela	29	29	203	7.000	0.241	[12]
Bridge Brook Lake	75	25	107	4.280	0.171	[13]
Burguess Shale (Cambrian)	48	48	249	5.188	0.108	[14]
Chengjiang (Cambrian)	33	33	99	3.000	0.091	[14]
Chesapeake Bay	33	31	72	2.323	0.075	[15]
Coachella Valley	30	29	262	9.034	0.312	[16]
Messel forest (Eocene)	700	630	5534	8.784	0.014	[10]
Messel full (Eocene)	700	700	6444	9.206	0.013	[10]
Messel full (Eocene) Red.	700	630	4602	7.305	0.012	[10]
Messel lake (Eocene)	94	94	517	5.500	0.059	[10]
Marine 2	557	557	126501	227.111	0.408	[22]***
Marine 2 Bathymetry	557	557	95989	172.332	0.309	[22]***
Reef 1	50	50	556	11.120	0.222	[17]
Reef 2	250	250	3355	13.420	0.054	[17]
Skipwith Pond	35	25	197	7.880	0.315	[18]
St. Marks Seagrass	48	48	221	4.604	0.096	[19]
St. Martin Island	44	42	205	4.881	0.116	[20]
Stony Stream	112	98	832	8.490	0.087	[21]
Ythan Estuary 1	134	129	598	4.636	0.036	[22]
Ythan Estuary 2	93	92	421	4.576	0.050	[23]
Scotch Broom	154	153	370	2.418	0.016	[24]
Mirror Lake	586	172	4322	25.128	0.146	[25]
El Verde Rainforest	156	146	1510	10.342	0.071	[26]
Maspalomas Lagoon	17	17	29	1.706	0.100	[27]
Hokkaido Forest	74	74	374	5.054	0.068	[28]
UK Grassland	75	66	113	1.712	0.026	[29]
Lake Tahoe	800	172	3885	22.587	0.131	[25]
Little Rock Lake	181	170	2375	13.971	0.082	[30]
Marine	67	67	601	8.970	0.134	[31]
Canton Creek	108	100	708	7.080	0.071	[21]
Prairie, Manitoba	15	15	27	1.800	0.120	[32]
Willow forest, Manitoba	12	12	18	1.500	0.125	[32]
Aspen, Manitoba	24	24	37	1.542	0.064	[32]
Aspen forest, Manitoba	32	32	56	1.750	0.055	[32]
Wytham Wood, UK	22	22	39	1.773	0.081	[32]
Salt Meadow, N-Z	32	32	35	1.094	0.034	[32]
Rain forest, Malaysia	11	11	15	1.364	0.124	[32]
Sand beach, California	14	14	23	1.643	0.117	[32]
Trelease Woods, Illinois	29	29	61	2.103	0.073	[32]
Montane forest, Arizona	33	33	69	2.091	0.063	[32]
Barren land, Spitsbergen	8	8	10	1.250	0.156	[32]
Pasture, Spitsbergen	11	11	12	1.091	0.099	[32]
Sand beach, South Africa	21	21	36	1.714	0.082	[32]
Old field, New Jersey	22	22	39	1.773	0.081	[32]
Shigayama forest, Japan	10	10	13	1.300	0.130	[32]
Alpine tundra, Montana	26	26	70	2.692	0.104	[32]
Tundra Prudhoe, Alaska	10	10	12	1.200	0.120	[32]
Yamal Peninsula, Siberia	11	11	17	1.545	0.140	[32]
Sand dunes, Namib desert	17	17	39	2.294	0.135	[32]
Rajasthan Desert, India	22	22	59	2.682	0.122	[32]
Lough Ine rapids, Ireland	9	9	13	1.444	0.160	[32]
Moosehead Lake, Maine	17	17	32	1.882	0.111	[32]
Nyasa lake, Malawi	31	31	95	3.065	0.099	[32]
Lake Texoma, Texas	19	19	68	3.579	0.188	[32]
Lake Rybinks, Russia	16	16	32	2.000	0.125	[32]
Heney Lake, Quebec	17	17	32	1.882	0.111	[32]
Hafner Lake, Austria	10	10	15	1.500	0.150	[32]
Finstertaler Lake, Austria	9	9	14	1.556	0.173	[32]
Neusiedler Lake, Austria	14	14	17	1.214	0.087	[32]
Lake Abaya, Ethiopia	13	13	24	1.846	0.142	[32]
Lake George, Uganda	16	16	27	1.688	0.105	[32]
Lake Piijirvi, Finland	21	21	29	1.381	0.066	[32]
Piijirvi littoral, Finland	27	27	70	2.593	0.096	[32]
Lake PyhajiU-vi, Finland	25	25	67	2.680	0.107	[32]
Crocodile Creek, Malawi	29	29	48	1.655	0.057	[32]
River Clydach, Wales	12	12	27	2.250	0.188	[32]
Morgan's Creek, Kentucky	13	13	36	2.769	0.213	[32]
River Rheidol, Wales	18	18	75	4.167	0.231	[32]
Yoshino River, Japan	13	13	25	1.923	0.148	[32]
River Thames, UK	10	10	18	1.800	0.180	[32]
Mississippi mudflats, Iowa	21	21	62	2.952	0.141	[32]
Crystal River, Florida	14	14	28	2.000	0.143	[32]
Lestijoki rapids, Finland	16	16	42	2.625	0.164	[32]
Average values	*100.2*	*82.27*	*3600*	*9.648*	*0.121*	

Table C. Basic topological descriptors (empirically measured) of different, representative, real trophic networks. The column "Ref." contains the original references from which all these values (or at least S and L) have been extracted (see References below). Asterisks (***) denote main text reference.

Spare nodes:

If spare nodes appeared, the network connectivity C was increased by a factor of 0.1 and the trophic preferences of the spare species relaxed by randomly assigning them broader trophic categories. This procedure was repeated until a fully connected network was generated, or until C reached the maximum empirically measured value 0.41 (Table C). If disconnected nodes persisted, they were removed, keeping the proportion of spare nodes (PSP) prior to fixation as a measure of data self-consistency (in an exhaustively sampled ecosystem, PSP=0).

Modularity analysis:

Modularity quantifies the extent to what a network can be divided into densely connected semi-independent modules (see S1 Text references [33,34] below). Using the Louvain method to find the optimal division that maximizes the number of within-module connections while minimizing interactions among different modules (see S1 Text reference [35]), we calculated, for each ecosystem, the maximal modularity (MOD), and the number of partitions (PART) and clustering coefficient (CLUST) under that maximal modularity (see Box-1 in the main Text).

Supplementary results:

Anomalous interactions: Unlikely interactions observed in some replicates help identifying potential sources of noise, but also buffering strategies.

In a small number of replicates, we found interactions that are very unlikely to occur in nature (for instance, interactions between species that do not share habitat such as a sea anemone eating a mouse). These interactions occur because the basic algorithm assumes every species coexist in space and time. To discriminate between sub-habitats or other constraining aspects of the ecology, trophic network should be unfolded in different dimensions, each corresponding to one ecological compartment (see ref. [40] in the main text). Fortunately, this procedure is also amenable for algorithmic automatization (see section on multilayer extensions).

Other anomalous interactions arise from the inclusion of anecdotal evidence in the databases (e.g., record size, diet in captivity). This is partially alleviated by the log (size) transformation and the coarse-graining algorithms (see Methods, Fig 6 and S2 Fig), though also raises a concern about the data quality in public databases (see Discussion). Finally, because of the coarse-graining in dietary attributes, some super-specialized taxa appear sometimes as less-generalist species preferring preys that differ from that found in nature. This over-generalization is counter-balanced by generalist species, which eat in each replicate a subset of all their potential preys.

The proportion of primary producers only affects some topological properties:

In our simulations, the trophic relationship between herbivores and primary producers are established randomly (that is, plants can be potentially consumed by any herbivore, with a probability C). Although this might seem an oversimplification, this choice is supported by three facts: 1) As a general rule, herbivores do not generally consume the whole plant. Because of this, the allometric relationship between plants and herbivores is either weak or nonexistent (consider for instance aphids feeding on a tree's fluids, and see ref. [37] in the main text). 2) By using a random assignation, the expected outcome will be a Binomial distribution of a form B~(N×BAS,C). That implies that although most plants will be consumed, on average, by N×BAS herbivores; there will also be, with lower probability, a number of highly specialized and truly generalist plants. According to the Central Limit Theorem (CLT), these specialized and generalist plant-herbivore interactions will in turn be connected, with some probability, to specialized and generalist predator-prey interactions (the later determined by allometric relationships, see Methods), therefore mimicking the specialist/generalist balance found in real networks (see ref. [32] in the main text). 3) Despite significant differences in the proportion of primary producers respect to the whole number of species (the descriptor BAS, see Box-1), which may span one order of magnitude, the proportion of primary producers only affects some of the considered topological descriptors (see S1 Fig):

Preliminary analysis of robustness against loses in data quality:

To address whether the realism of our automatically generated trophic networks relies more on the allometric niche model itself or on the quality and density of the species attributes recorded, we performed two sensitivity analyses: First, we introduced a proportion of noisy (random) data in the species' body size, diet or both. Second, we replaced a proportion of the original body sizes or dietary attributes by arbitrary fixed values (targeted biases). If distortion in body sizes changes dramatically the network topology, then the realism of our networks can be attributed to the ability of the allometric niche model to create plausible topologies from relative body sizes alone. If, contrarily, the topology changes more abruptly when the dietary preferences are altered, then the realism of our networks would emerge from diet-taxon matching.

Our results, summarized in S2 Fig, suggest that, under random perturbations, our algorithm is still capable of generating topologically realistic networks. Indeed, it converges, under randomization, to the pure niche model (see panel A in S1 Fig), and most network descriptors remain similar. However, the identity of nodes as species that occupy realistic positions within the network (which is a distinctive feature of our approach, see above) can disappear completely when most species are assigned a random size or diet. In other words, while the niche model is mainly involved in creating the network architecture, the diet-taxon matching criterion is involved in realistically sorting each species within that network architecture.

S1 Text (Supplementary) bibliographical references:

1. Robinson LA, Greenstreet SPR, Reiss H, Callaway R, Craeymeersch JAM, de Boois IJ, Degraer S, Ehrich S, Fraser HM, Goffin A, Kröncke I, Lindal Jorgenson L, Robertson MR, Lancaster J. Length-weight relationships of 216 North Sea benthic invertebrates and fish. *J Mar Biol Assoc UK.* 2010;90(1):95-104.
2. Gowing G, Recher HF. Length-weight relationships for invertebrates. *Aust J Ecol*. 1984;9(1):5-8.
3. Ganihar SR. Biomass estimates of terrestrial arthropods based on body length. *J Biosci.* 1997;22(2):219-224.
4. Santini L, Benítez-López A, Ficetola GF, Huijbregts MAJ. Length-mass allometries in amphibians. *Integr Zool*. 2018;13(1):36-45.
5. Meiri S. Length-weight allometries in lizards. *J Zool.* 2010;281(3):218-226.
6. Silva M. Allometric scaling of body length: elastic or geometric similarity in mammalian design. *J Mammal.* 1998;79(1): 20-32.
7. Hardin G. The competitive exclusion principle. *Science.* 1960;131(3409):1292-1297.
8. Büchi L, Vuilleumier S. Coexistence of specialist and generalist species is shaped by dispersal and environmental factors. *Am Nat.* 2014;183(5):612-624.
9. Halnes G, Fath BD, Liljenström H. The modified niche model: Including detritus in simple structural food web models. *Ecol Model.* 2007;208(1):9-16.
10. Dunne JA, Labandeira CC, Williams RJ. Highly resolved early Eocene food webs show development of modern trophic structure after the end-Cretaceous extinction. *Proc R Soc B.* 2014;281:1782.
11. Warren CP, Pascual M, Lafferty KD, Kuris AM. The inverse niche model for food webs with parasites. *Theor Ecol.* 2010;3(1):285-294.
12. Yodzis P. Local trophodynamics and the interaction of marine mammals and fisheries in the Benguela ecosystem. *J Anim Ecol.* 1998;67:635-658.
13. Havens K. Scale and structure in natural food webs. *Science.* 1992;257(5073):1107-1109.
14. Dunne JA, Williams RJ, Martinez ND. Compilation and network analyses of Cambrian food webs. *PLoS Biol.* 2008;6(4):e102.
15. Baird D, Ulanowicz RE. The seasonal dynamics of the Chesapeake Bay ecosystem. *Ecol Monogr.* 1989;59(4):329-364.
16. Polis GA. Complex trophic interactions in deserts: an empirical critique of food-web theory. Am Nat. 1991;138(1):123-155.
17. Opitz S. Trophic interactions in Caribbean coral reefs. *ICLARM Technical Reports*, 43, pp. 341. Manila, Philippines. 1996.
18. Warren PH. Spatial and temporal variation in the structure of a freshwater food web. *Oikos*. 1989;55(3):299-311.
19. Christian RR, Luczkovich JJ. Organizing and understanding a winter's seagrass food web through effective trophic levels. *Ecol Model.* 1999;117(1):99-124.
20. Goldwasser L, Roughgarden J. Construction and analysis of a large Caribbean food web. *Ecology.* 1993;74(4):1216-1233.
21. Townsend CR, Thompson RM, McIntosh AR, Kilroy C, Edwards E, Scarsbrook MR. Disturbance, resource supply, and food-web architecture in streams. *Ecol Lett*. 1998;1:1461-0248.
22. Hall SJ, Raffaelli D. Food-web patterns: lessons from a species-rich web. *J Anim Ecol.* 1991;60(3):823-841.
23. Huxham M, Beaney S, Raffaelli D. Do parasites reduce the chances of triangulation in a real food web? *Oikos*. 1996;76(2):284-300.
24. Memmott J, Martinez ND, Cohen JE. Predators, parasitoids and pathogens: species richness, trophic generality and body sizes in a natural food web. *J Anim Ecol.* 2000;69(1):1-15.
25. Dunne JA, Williams RJ, Martinez ND. Food-web structure and network theory: the role of connectance and size. *Proc Natl Acad Sci USA.* 2002;99(20):12917-12922.
26. Reagan DP, Waide RB, editors. *The food web of a tropical rain forest.* Chicago: University of Chicago Press, Chicago. 1996.
27. Almunia J, Basterretxea G, Arístegui J, Ulanowicz RE. Benthic-pelagic switching in a coastal subtropical lagoon. *Estuar Coast Shelf Sci.* 1999;49(3):363-384.
28. Hirao T, Murakami M. Quantitative food webs of lepidopteran leafminers and their parasitoids in a Japanese forest. *Ecol Res.* 2008;23(1):159-168.
29. Martinez ND, Hawkins BA, Dawah HA, Feifarek BP. Effects of sampling effort on characterization of food-web structure. *Ecology.* 1999;80(3):1044-1055.
30. Martinez ND. Artifacts or attributes? Effects of resolution on the Little Rock Lake food web. *Ecol Monogr*. 1991;61(4):367-392.
31. Brose U, Cushing L, Berlow EL, Jonsson T, Banasek-Richter C, Bersier LF, Blanchard JL, Brey T, Carpenter SR, Blandenier MFC, Cohen JE. Body sizes of consumers and their resources. *Ecology.* 2005;86(9):2545.
32. Vitekere K. Complexity, connectance and link density in continental food webs: dissimilarities in aquatic and terrestrial food webs and their habitats. *Appl Ecol Environ Res.* 2021;19:817-831.
33. Gilpin M, editor. *Metapopulation dynamics: empirical and theoretical investigations*. San Diego: Academic Press; 2012.
34. Newman MEJ, Girvan M. Finding and evaluating community structure in networks. *Phys Rev E.* 2004;69(2):026113.
35. Blondel VD, Guillaume JL, Lambiotte R, Lefebvre E. . Fast unfolding of communities in large networks. *JJ Stat Mech Theory Exp.* 2008; *2008*(10):P10008.
